# Investigation of Anti-Liver Cancer Activity of the Herbal Drug FDY003 Using Network Pharmacology

**DOI:** 10.1155/2022/5765233

**Published:** 2022-09-09

**Authors:** Ho-Sung Lee, In-Hee Lee, Sang-In Park, Minho Jung, Seung Gu Yang, Tae-Wook Kwon, Dae-Yeon Lee

**Affiliations:** ^1^The Fore Research Institute, 33 Saemunan-ro 5ga-gil, Jongno-gu, Seoul 03170, Republic of Korea; ^2^Forest Hospital, 33 Saemunan-ro 5ga-gil, Jongno-gu, Seoul 03170, Republic of Korea; ^3^Forestheal Hospital, 173 Ogeum-ro, Songpa-gu, Seoul 05641, Republic of Korea; ^4^Forest Hospital, 129 Ogeum-ro, Songpa-gu, Seoul 05549, Republic of Korea; ^5^Forest Hospital, 67 Dolma-ro, Bundang-gu, Seongnam 13586, Republic of Korea

## Abstract

Globally, liver cancer (LC) is the sixth-most frequently occurring and the second-most fatal malignancy, responsible for 0.83 million deaths annually. Although the application of herbal drugs in cancer therapies has increased, their anti-LC activity and relevant mechanisms have not been fully studied from a systems perspective. To address these issues, we conducted a system-perspective network pharmacological investigation into the activity and mechanisms underlying the action of the herbal drug. FDY003 reduced the viability of human LC treatment. FDY003 reduced the viability of human LC cells and elevated their chemosensitivity. There were a total of 16 potential bioactive chemical components in FDY003 and they had 91 corresponding targets responsible for the pathological processes in LC. These FDY003 targets were functionally involved in regulating the survival, proliferation, apoptosis, and cell cycle of LC cells. Additionally, we found that FDY003 may target key signaling cascades connected to diverse LC pathological mechanisms, namely, PI3K-Akt, focal adhesion, IL-17, FoxO, MAPK, and TNF pathways. Overall, this study contributed to integrative mechanistic insights into the anti-LC potential of FDY003.

## 1. Introduction

Liver cancer (LC) is the sixth-most frequently occurring and the second-most fatal malignancy, responsible for 0.83 million deaths annually [1]. Molecular-targeted and immune checkpoint therapies serve as the main treatment strategies for LC in clinical settings [2]. However, such treatment options and their efficacy are largely restricted to a few patients, which emphasizes the need to design and develop effective therapeutics for LC treatment [2]. There is growing recognition of herbal drugs as potent therapeutics for LC treatment to augment treatment efficacy while minimizing the adverse effects of anticancer strategies [3–7]. Herbal drugs possess beneficial properties, increasing survival and tumor response rates, enhancing the treatment efficacies and immune functions, increasing the quality of life, and inhibiting the development of side effects caused by anticancer treatments in patients with LC [3–7].

FDY003 is an herbal drug composed of *Cordyceps militaris* (Cm), *Artemisia capillaris* Thunberg (AcT), and *Lonicera japonica* Thunberg (LjT). It has antiproliferative and proapoptotic activities in cancer cells [8–11]. These anticancer activities arise primarily from the functional regulation of many oncogenes, tumor suppressors, and their cancer-associated signaling cascades [8–11]. However, the anticancer activity of FDY003 against LC and its mechanistic characteristics from an integrated systems view remain unclear.

Network pharmacology is a field that is efficient for dissecting synergistic mechanistic characteristics of herbal drugs based on their related pharmacological and biomedical data [12–19]. This strategy merges the herbal drug-associated comprehensive information into networks that represent the interaction between herbal medicines, bioactive components, and therapeutically targeted genes and proteins [12–19]. Network pharmacology seeks the synergistic mechanisms of herbal drugs by assessing the functional, structural, and topological nature of their associated networks [12–19]. Based on the network pharmacology, we assessed the therapeutic role of FDY003 in LC and evaluated the underlying systematic mechanisms.

## 2. Materials and Methods

### 2.1. Cell Culture

The HepG2 human LC cell line was obtained from the Korean Cell Line Bank (Seoul, Korea) and cultured in Dulbecco's modified Eagle medium (WELGENE Inc., Gyeongsang, Korea) supplemented with antibiotics (penicillin-streptomycin) (Thermo Fisher Scientific, Inc., Waltham, MA, USA) and 10% fetal bovine serum (FBS) (WELGENE Inc.). The cells were grown in a humidified incubator at 37°C with 5% CO_2_.

### 2.2. Herbal Drug Preparation

For the preparation of FDY003, dried raw AcT (150.0 g), LjT (150.0 g), and Cm (100.0 g) were obtained from Hanpure Pharmaceuticals (Pocheon, Korea), mixed, and ground. The herbal mixture was dispersed in 70% ethanol (500 mL) and subjected to a 3 h reflux extraction process at −80°C. After purifying the herbal extracts with 80% and 90% ethanol solutions successively, they were lyophilized at −80°C and the resulting 50.4 g of freeze-dried extracts were stored at −20°C. We used distilled water to dissolve the prepared FDY003 samples before further experiments.

### 2.3. Analysis of Cell Viability upon Drug Treatment

The viability of the drug-treated cells was analyzed by water-soluble tetrazolium salt (WST-1) experiments. Cells (1.0 × 10^4^) were seeded and cultured in 96-well plates and treated with FDY003, sorafenib (Sigma-Aldrich, St. Louis, MO, USA), or both for 72 h. Next, cells were incubated for 2 h with WST-1 solution (Daeil Lab Service Co. Ltd., Seoul, Korea) at 37°C and 5% CO_2_. The cell viability was assessed by measuring the absorbance (450 nm) of the samples on an xMark microplate absorbance spectrophotometer (Bio-Rad, Hercules, CA, USA).

### 2.4. Screening for Bioactive Chemical Components

First, we investigated comprehensive herbal medicine-related databases (e.g., a bioinformatics analysis tool for the molecular mechanism of traditional Chinese medicine [20], an anticancer herbs database of systems pharmacology [21], and the traditional Chinese medicine systems pharmacology [22]) and acquired large-scale data for the chemical composition of FDY003. For these components, we used pharmacokinetic parameters, namely drug-likeness, Caco-2 permeability, and oral bioavailability, for the screening of the bioactive chemical components, as previously described [14, 22, 23]. Drug-likeness determines whether a given chemical component can adequately serve as a drug based on its molecular structure and physicochemical properties [22, 24]. A chemical component with a drug-likeness equal to or greater than 0.18 (the mean drug-likeness based on the currently available drugs) is considered to have adequate drug-like functions [22, 24]. The Caco-2 permeability determines whether a given chemical component has adequate intestinal absorption and permeability [22, 25–27]; a chemical component with Caco-2 permeability equal to or greater than −0.4 is considered to have adequate intestinal permeability and absorption activity [28, 29]. Oral bioavailability determines whether an orally administered chemical component has an adequate delivery rate to reach the target sites of drug action [22, 30]. A chemical component with an oral bioavailability equal to or more than 30% is considered to have adequate absorption in the human body [22, 30]. Thus, chemical components with drug-likeness ≥ 0.18, Caco-2 permeability ≥ −0.4, and oral bioavailability ≥ 30% [14, 22, 23] were considered bioactive.

### 2.5. Target Screening

To screen the targets of the identified bioactive chemical components of FDY003, we investigated their simplified molecular input line entry system notation using PubChem [28] and imported the information into the diverse tools and databases used to evaluate the molecular interactions between the chemical components and genes and proteins as follows: PharmMapper [29], Similarity Ensemble Approach [31], SwissTargetPrediction [32], and Search Tool for Interactions of Chemicals 5 [33]. Among the FDY003 targets, the genes and proteins significantly associated with the LC pathological mechanisms were examined using “liver cancer” and “hepatocellular carcinoma” as query keywords in the following tools and databases: Pharmacogenomics Knowledgebase [34], Online Mendelian Inheritance in Man [35], Human Genome Epidemiology Navigator [36], DisGeNET [37], Comparative Toxicogenomics Database [38], Therapeutic Target Database [39], GeneCards [40], and DrugBank [41].

### 2.6. Generation and Analysis of Herbal Drug-Associated Networks

Herbal drug-associated networks consist of nodes (which refer to the herbal components, chemical components, targeted genes and proteins, and pathways) and edges (which refer to the interactions among the network nodes) [42]. The node degree represents the number of edges for a given node [42]. The herbal component–chemical component–target (H-C-T) network uses herbal components, chemical components, and targeted genes and proteins of FDY003 as nodes and their molecular interactions and associations as the edges. The H-C-T-Pathway (H-C-T-P) network was created by linking the targets of the H-C-T network to their correspondingly enriched LC-related signaling cascades. The protein-protein interaction (PPI) network used FDY003 targets as nodes and their interaction information (interactions with high confidence obtained from STRING [43]) as edges. Network generation and analysis were performed using the Cytoscape tool [44].

### 2.7. Analysis of Correlation between the Survival of Patients with LC and the Expression Profiles of FDY003 Targets

The correlations between the survival of patients with LC and the expression profiles of LC-associated targets of FDY003 were analyzed using the Kaplan–Meier Plotter [45], a commonly used database for the survival analysis with large-scale gene expression profiles and survival information of patients with various cancer types obtained from The Cancer Genome Atlas (TCGA) [46], the Gene Expression Omnibus (GEO) [47], and the European Genome-phenome Archive (EGA) [48]. The survival analysis was performed using an auto-selected best cut-off and the results with *p* < 0.05 (log-rank test) were regarded to be statistically significant. The clinical information of the included patients for the survival analysis is provided in Supplementary Table S1.

### 2.8. Exploration of Functional Enrichment of FDY003 Targets

The Gene Ontology (GO) and pathway enrichment of FDY003 targets were analyzed using the Database for Annotation, Visualization, and Integrated Discovery (DAVID) [49] and the Kyoto Encyclopedia of Genes and Genomes (KEGG) [50], respectively, and the results having *p* < 0.05 were regarded to be statistically significant.

### 2.9. Investigation of Binding Affinities between the Chemical Components and Targets of FDY003

To assess the binding affinities between the chemical components and corresponding targets of FDY003, we calculated the binding energies. First, we imported the molecular structures of the bioactive components of FDY003 (obtained from the RCSB Protein Data Bank [51]) and their targets (obtained from PubChem [28]) into AutoDock Vina [52] and calculated the resulting binding energies. As suggested earlier [53, 54], the binding affinities between the chemical components and targets were considered to be highly significant if their corresponding binding energies were ≤ −5.0.

## 3. Results

### 3.1. Inhibitory Role of FDY003 in LC

To assess the action of FDY003 against LC, HepG2 human LC cells were treated with FDY003 and/or sorafenib (a first-line therapeutic agent for LC treatment [2]), and the effects of the treatment on the cells were monitored. FDY003 significantly reduced the viability of HepG2 cells; the viability of the cells was further inhibited upon treatment with a combination of the herbal drug and sorafenib (Supplementary Figures S1A and S1B). These observations suggest that FDY003 exhibits inhibitory and chemosensitizing effects on the LC cells.

### 3.2. Identification of Bioactive Components and Targets of FDY003

**Figure 1:**
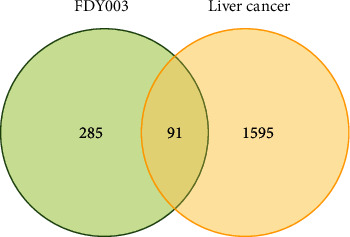
A Venn diagram of targets of FDY003 (green circle) and LC-associated genes (yellow circle).

As previously described, we selected FDY003 constituents with Caco-2 permeability ≥ −0.4, drug-likeness ≥ 0.18, and oral bioavailability ≥ 30%, and considered them as bioactive components (Supplementary Tables S2 and S3) [8, 14, 22, 23]. Several components having potent anticancer functions against LC were also included in the list of bioactive components, despite their failure to meet the inclusion criteria (Supplementary Tables S2 and S3). Using the databases for examining the molecular binding interactions between the chemical components and genes and proteins, a total of 379 targets were investigated. Among them, 91 were determined to be associated significantly with the LC pathological mechanisms based on previous cancer biology and oncological studies (Figure 1, and Supplementary Tables S4 and S5).

### 3.3. Network Analysis of Poly-Pharmacological Mechanisms Underlying FDY003 Effects in LC Treatment

**Figure 2:**
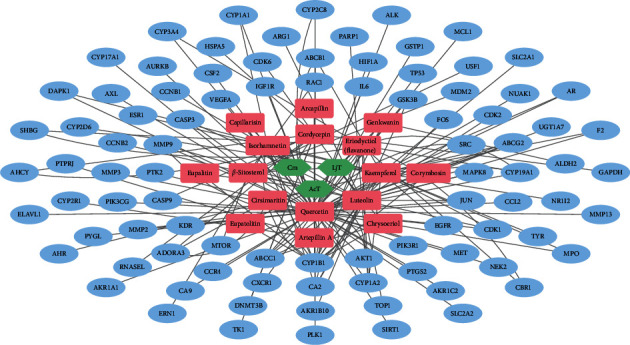
The herbal component–bioactive chemical component-target network for FDY003. Green nodes, herbal components; red nodes, bioactive chemical components; blue nodes, liver cancer-associated targets.

For the network pharmacology analysis, first, we united the FDY003-associated data and knowledge into an H-C-T network (Figure 2, and Supplementary Table S4 and S5). Among the targets in this network, 20.1% (19 of 91 targets), 23.1% (21 of 91 targets), and 74.7% (68 of 91 targets) were linked to the chemical components kaempferol, luteolin, and quercetin, respectively (Figure 2 and Supplementary Table S4), which demonstrated their key roles in exerting anti-LC effects of FDY003. Moreover, 70.3% (64 of 91 targets) were linked to two or more chemical components (Figure 2), which indicated the synergistic poly-pharmacological nature of the herbal drug.

**Figure 3:**
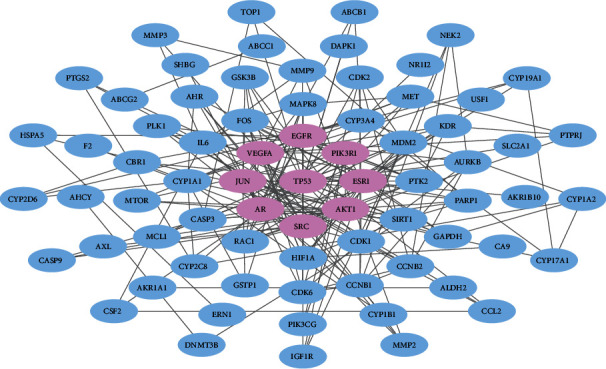
The protein-protein interaction network for liver cancer-associated targets of FDY003. Purple nodes, hubs.

**Figure 4:**
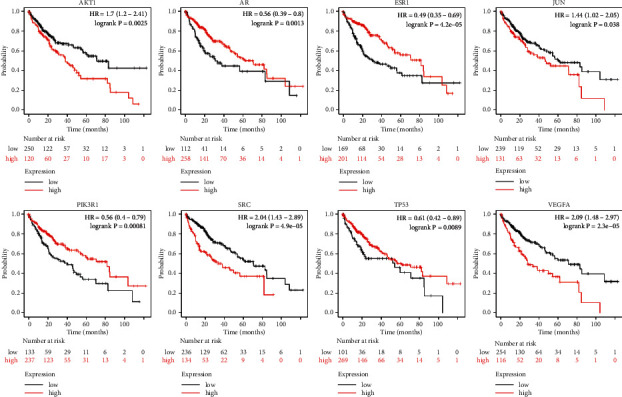
Analysis of the survival probability of patients for corresponding liver cancer-associated targets of FDY003. The Kaplan–Meier curves for the survival of liver cancer patients are associated with the expression levels of the indicated targets.

Analytical investigation of complex interactions among the therapeutic targets of a given drug is pivotal for understanding the underlying treatment mechanisms [55–59]. Thus, we constructed a PPI network consisting of large-scale interactions among the FDY003 targets to further dissect their pharmacological features (Figure 3). For analyzing the network topology of the PPI network, we identified the network hubs having a relatively large number of interacting partners as compared to the nonhubs; they had a high probability of being effective drug targets [60, 61]. As described previously, nodes with number of edges ≥ 2 × the average node degree were considered hubs [62, 63]. As a result, the identified hubs were AKT1, AR, EGFR, ESR1, JUN, PIK3R1, SRC, TP53, and VEGFA (Figure 3), which suggests that they were the primary nodes mediating the therapeutic roles of FDY003 in LC. Additionally, the expression status of these hubs could estimate the survival rate of patients with LC (Figure 4), which suggests their prognostic roles.

**Figure 5:**
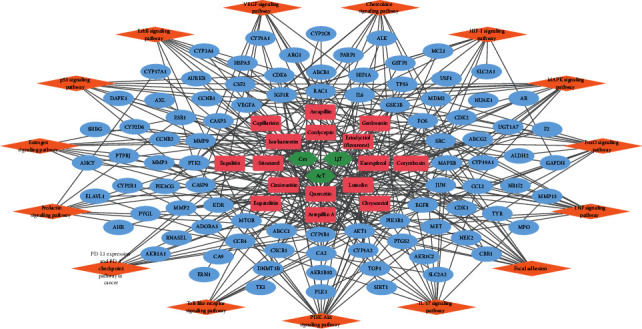
The herbal component–bioactive chemical component-target-pathway network for FDY003. Green nodes, herbal components; red nodes, bioactive chemical components; blue nodes, liver cancer-associated targets; orange nodes, liver cancer-associated pathways.

To understand the mechanisms of action of FDY003 in LC treatment, we evaluated the GO terms and pathways that were functionally enriched for the targets. The results showed that the FDY003 targets were functionally involved in the control of survival and proliferative behaviors, apoptosis, and cell cycle of LC cells (Supplementary Figure S2), which suggests the potential FDY003 mechanisms in the LC treatment. Additionally, FDY003 could target key signaling cascades connected to diverse LC pathological mechanisms (Figure 5 and Supplementary Figure S2) as analyzed by the KEGG pathway enrichment investigation, which demonstrated that these pathways were important mediators of the FDY003 drug activity against LC.

### 3.4. Assessing the Binding Affinities of Chemical Components of FDY003 and Their Targets

**Figure 6:**
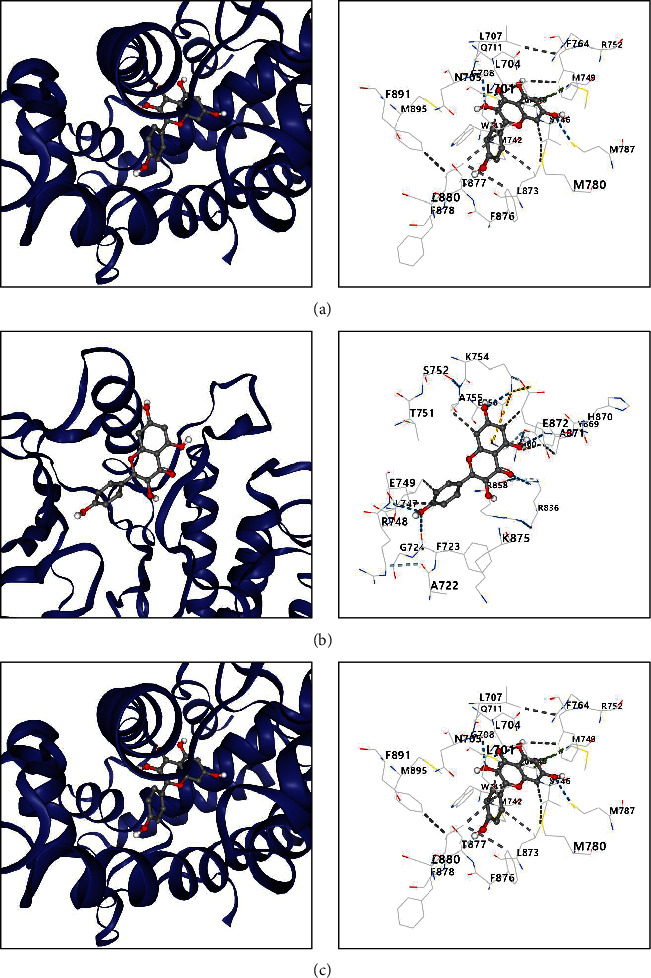
Analysis of binding affinities between the bioactive chemical components of FDY003 and their targets. (a) Kaempferol–AR (binding energy = −8.0 kcal/mol). (b) Kaempferol–EGFR (binding energy = −8.1 kcal/mol). (c) Kaempferol–ESR1 (binding energy = −8.6 kcal/mol).

We performed a molecular docking study to assess the binding affinities and active binding sites between the chemical components of FDY003 and their targets. The resulting binding energies of the chemical component-target pairs were less than −5.0 kcal/mol (Figure 6 and Supplementary Figure S3 and Supplementary Table S6), which indicated their high pharmacological binding potentials.

## 4. Discussion

Globally, LC is the sixth-most frequently occurring and the second-most fatal malignancy, responsible for 0.83 million deaths annually [1]. Although the application of herbal drugs in cancer therapies has increased, their anti-LC activity and relevant mechanisms have not been fully studied from a systems view [3–7]. To address these issues, we conducted a system-perspective network pharmacological investigation into the activity and mechanisms of action of the herbal drug, FDY003, for LC treatment. Treatment with FDY003 significantly reduced the viability of human LC cells and elevated their chemosensitivity. A total of 16 potential bioactive chemical components of FDY003 and corresponding 91 targets were identified for the pathological process of LC. The FDY003 targets were functionally involved in the regulating survival, proliferation, apoptosis, and cell cycle of LC cells. Additionally, we found that FDY003 may target key signaling cascades connected to the diverse LC pathological mechanisms, namely, phosphoinositide 3-kinase (PI3K)-Akt, focal adhesion, interleukin (IL)-17, forkhead box O (FoxO), mitogen-activated protein kinase (MAPK), and tumor necrosis factor (TNF) pathways. Overall, this study added to the integrative mechanistic insights into the anti-LC potential of FDY003.

The hub targets identified from the analysis of the FDY003-associated PPI network modulate crucial LC pathological mechanisms, and this targeting may result in effective therapeutic effects against LC. *AKT1* is an important regulator of the tumorigenic processes, migration, spreading, therapeutic sensitivity, resistance, survival, and proliferation of LC cells, and is a promising theragnostic target [64–67]. *AR* expression and activity influence angiogenesis, stemness, invasion, angiogenesis, epithelial-mesenchymal transition (EMT), migration, and oncogenesis of LC cells, and it is a prognostic determinant and responsive marker for anticancer therapies [68–73]. *EGFR* is responsible for the modulation of proliferation, metastasis, migration, self-renewal potential, EMT, angiogenesis, anchorage-independent growth, invasion, and drug sensitivity of LC cells and tumors, and its expression and activity are implicated in the initiation, recurrence, progression, metastasis, and aggressiveness of LC in patients [74–83]. *ESR1* is involved in the coordination of migration, angiogenesis, cancer stem-like properties, mobility, proliferation, and invasion of LC cells, and its genetic and expression status correlate with cancer susceptibility, tumor growth, metastasis, and prognosis of LC [84–89]. *JUN* is an oncogenic transcription factor that promotes tumor formation, chemoresistance, invasion, proliferation, migration, and metastasis, and serves as a marker for evaluating chemotherapeutic responses [90–92]. *PIK3R1* is highly expressed and activated in LC cells and tumors, and it stimulates their migration, proliferation, invasion, and survival [93, 94]. Its elevated expression level is associated with poor prognostic outcomes [93, 94]. *Src* is highly expressed in LC cells and tumors, and its targeting can repress anoikis resistance, metastasis, treatment resistance, growth, stemness, invasion, tumorigenesis, migration, and mobility [95–101]. The expression and mutations of *TP53* serve as biomarkers of prognosis, therapeutic response, aggressiveness, progression, and survival in LC [102–107]. *VEGFA* contributes to the malignant processes in LC by inducing angiogenesis, growth, invasion, prosurvival, metastasis, lymphangiogenesis, and migration of LC cells and tumors, and its expression and polymorphisms are related to the therapeutic responsiveness, prognosis, and cancer susceptibility of patients with LC [108–113].

FDY003 may pharmacologically intervene in various signaling cascades that are key factors in the LC pathophysiology as well as potent treatment targets. The chemokine pathway impacts the pathological processes of LC in various aspects, including inflammation, immune response, angiogenesis, metastasis, invasion, carcinogenesis, migration, EMT, tumorigenic potential, tumor microenvironment remodeling, and proliferation of LC cells and tumors [114–117]. Its activity is further associated with decreased survival and poor prognosis in patients with LC [114–117]. The abnormally controlled function of the erythroblastic leukemia viral oncogene homolog (ErbB), focal adhesion, hypoxia-inducible factor (HIF)-1, MAPK, p53, PI3K-Akt, and vascular endothelial growth factor (VEGF) pathways may contribute to the malignant tumorigenic and progressive processes in LC by inducing uncontrolled survival and proliferation, angiogenesis, EMT, invasion, migration, and metastasis of LC cells and tumors [118–121]. Thus, they can function as key therapeutic targets [118–121]. The estrogen pathway exerts a protective role against LC by inhibiting cancerous inflammation [122, 123]. The FoxO pathway is a tumor-suppressing cascade whose activation induces antiproliferation and apoptosis of LC cells and confers the pharmacological effects of anticancer therapeutics [124–127]. The inflammatory IL-17, TNF, and toll-like receptor pathways are responsible for the modulation of not only the protumorigenic inflammation but also the proliferation, invasion, metastasis, migration, immune microenvironment, and treatment sensitivity of LC cells and tumors [128–134]. Their activities correlate with poor prognostic outcomes in patients with LC [128–134]. The programmed death-ligand 1 (PD-1)/programmed cell death-ligand 1 (PD-L1) pathway drives the immune escape of LC cells and tumors and is the major target of immune checkpoint therapies [135, 136]. The activity of the prolactin pathway is enhanced in LC tissues and is a marker for disease stage, survival, and progression of LC [137–140].

The herbal and bioactive components of FDY003 are known to function as anti-LC pharmacological agents. AcT represses survival, invasion, growth, angiogenesis, and migration of LC cells by blocking the PI3K/Akt and IL-6/signal transducer and activator of transcription (STAT) 3 pathways [141–144]. Cm exerts antisurvival, antigrowth, and antiangiogenic effects in LC cells [145, 146]. Cordycepin targets integrin, focal adhesion kinase (FAK), c-Jun N-terminal kinases (JNK), PI3K/Akt/mammalian target of rapamycin (mTOR), nuclear factor erythroid-2-related factor 2 (Nrf2)/heme oxygenase 1 (HO-1)/nuclear factor kappa-light-chain-enhancer of activated B cells (NF-*κ*B), IL-6, IL-1*β*, TNF-*α*, extracellular-signal-regulated kinase (ERK), Fas, B-cell lymphoma-2 (Bcl-2), caspase, and C-X-C motif chemokine receptor (CXCR) 4 cascades, thereby leading to the repression of angiogenesis, metastasis, EMT, proliferation, viability, and invasion of LC cells [147–153]. Eriodyctiol induces cell cycle arrest and apoptosis of LC cells by targeting the poly ADP-ribose polymerase (PARP), Bcl-2, and BCL2-associated X (Bax) signaling [154]. Isorhamnetin and *β*-sitosterol suppress the survival, viability, and proliferation of LC cells [155, 156]. Kaempferol enhances the efficacy of anticancer agents whilst reducing the proliferative, migratory, and invasive behaviors of LC cells by modulating PI3K/mTOR/matrix metalloproteinase (MMP), endoplasmic reticulum (ER) stress/CCAAT/enhancer binding protein (CHOP)/autophagy, 5′ adenosine monophosphate-activated protein kinase (AMPK), reactive oxygen species (ROS), and caspase pathways [157–161]. Luteolin induces autophagy, cell cycle arrest, growth suppression, chemosensitization, proapoptosis, anti-invasion, antimigration, antiadhesion, and antiangiogenesis effects on LC cells [162–170]. These pharmacological effects are conferred through cyclin, p53, JNK, death receptor, Akt/osteopontin, PARP, caspase, myeloid cell leukemia 1 (Mcl-1), X-linked inhibitor of apoptosis protein (XIAP), Beclin-1, Bcl-2, Bax, BH3 interacting domain death agonist (Bid), and the ER stress signaling pathways [162–170]. Quercetin represses chemoresistance, survival, growth, migration, and proliferation of LC cells by perturbing the activities of p53, B-cell lymphoma-extra-large (Bcl-xL), Janus kinase (JAK)/STAT, *β*-catenin, casein kinase (CK) 2*α*, Notch1, hedgehog, cyclin, PI3K/Akt, protein kinase C (PKC), cyclooxygenase-2 (COX-2), Bax, specificity protein 1 (SP1), and mitogen-activated protein kinase (MEK)/ERK pathways [171–180].

## 5. Conclusions

In conclusion, we performed a system-perspective network pharmacological investigation into the activity and mechanisms underlying the effects of the herbal drug, FDY003, for LC treatment. FDY003 could reduce the viability of human LC cells and elevate their chemosensitivity. A total of 16 potential bioactive chemical components of FDY003 that pharmacologically regulate diverse LC-related drug targets and signaling cascades were identified. Further studies should focus on enhancing the therapeutic potential of herbal drugs as efficacious clinical agents for anticancer treatment, including investigations into their mechanisms of action in the modulation of a variety of cancerous and protumorigenic phenotypes and therapeutic sensitivities.

## Data Availability

All data generated or analyzed during this study are included in this published article and its supplementary materials file.

## Supplementary Materials

Supplementary Materials Supplementary Figure S1. Pharmacological role of FDY003 on the viability of human liver cancer cells. Supplementary Figure S2. Gene ontology and pathway enrichment analysis for the liver cancer-related targets of FDY003. Supplementary Figure S3. Analysis of binding affinities between the bioactive chemical components of FDY003 and their targets. Supplementary Table S1. Clinical information of the included patients for the survival analysis. Supplementary Table S2. List of chemical components of FDY003. Supplementary Table S3. List of bioactive chemical components of FDY003. Supplementary Table S4. List of targets for active phytochemical ingredients of FDY003. Supplementary Table S5. List of the genes and proteins associated with the pathological mechanisms of liver cancer. Supplementary Table S6. Binding energies between the bioactive chemical components of FDY003 and their targets.
