# Supplementary material for: Investigation of Anti-Liver Cancer Activity of the Herbal Drug FDY003 Using Network Pharmacology
Source: Evid Based Complement Alternat Med. 2022 Sep 9;2022:5765233. doi: 10.1155/2022/5765233 (PMC9481369; doi:10.1155/2022/5765233)
Supplement: Supplementary Materials — Supplementary Figure S1. Pharmacological role of FDY003 on the viability of human liver cancer cells. Supplementary Figure S2. Gene ontology and pathway enrichment analysis for the liver cancer-related targets of FDY003. Supplementary Figure S3. Analysis of binding affinities between the bioactive chemical components of FDY003 and their targets. Supplementary Table S1. Clinical information of the included patients for the survival analysis. Supplementary Table S2. List of chemical components of FDY003. Supplementary Table S3. List of bioactive chemical components of FDY003. Supplementary Table S4. List of targets for active phytochemical ingredients of FDY003. Supplementary Table S5. List of the genes and proteins associated with the pathological mechanisms of liver cancer. Supplementary Table S6. Binding energies between the bioactive chemical components of FDY003 and their targets. [file 5765233.f1.docx]

**Supplementary Materials**

**Investigation of anti-** **liver cancer activity of the herbal drug FDY003
using network pharmacology**

Ho-Sung Lee^1,2,*^, In-Hee Lee^1,2^, Sang-In Park^3^, Minho Jung^4^, Seung Gu Yang^5^,
Tae-Wook Kwon^2^, and Dae-Yeon Lee^1,2,*^

^1^ The Fore Research Institute, 33 Saemunan-ro 5ga-gil, Jongno-gu, Seoul 03170, Republic of Korea.
^2^ Forest Hospital, 33 Saemunan-ro 5ga-gil, Jongno-gu, Seoul 03170, Republic of Korea.
^3^ Forestheal Hospital, 173 Ogeum-ro, Songpa-gu, Seoul 05641, Republic of Korea.
^4^ Forest Hospital, 129 Ogeum-ro, Songpa-gu, Seoul 05549, Republic of Korea.
^5^ Forest Hospital, 67, Dolma-ro, Bundang-gu, Seongnam 13586, Republic of Korea.

^*^Correspondence should be addressed to Ho-Sung Lee (forehslee@gmail.com)
and Dae-Yeon Lee (foresthrnd@gmail.com)

## Supplementary Figures


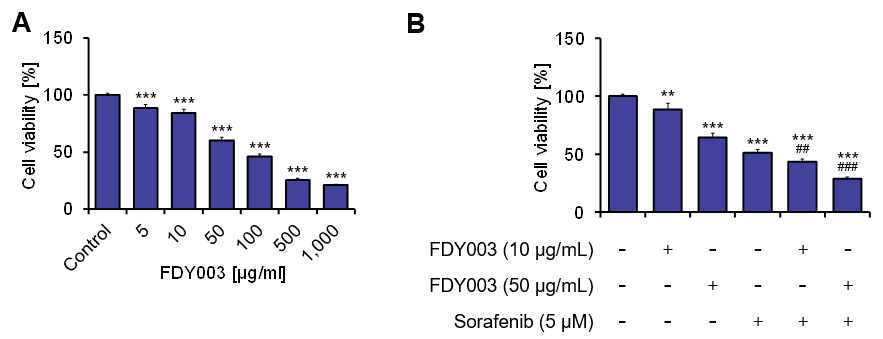


**Supplementary Figure S1. Pharmacological role of FDY003 on the viability of human liver cancer cells. (A)** HepG2 human liver cancer cells were treated with indicated doses of FDY003 and their viability was measured. **(B)** HepG2 cells were treated with indicated doses of FDY003 and/or sorafenib for 72 hours and their viability was measured. Data represent the mean ± S.E.M. of five replicates. **, p < 0.01; ***, p < 0.001; two-tailed Student’s t-test versus the untreated control cells. ##, p < 0.01; ###, p < 0.001; two-tailed Student’s t-test versus the sorafenib-treated cells.


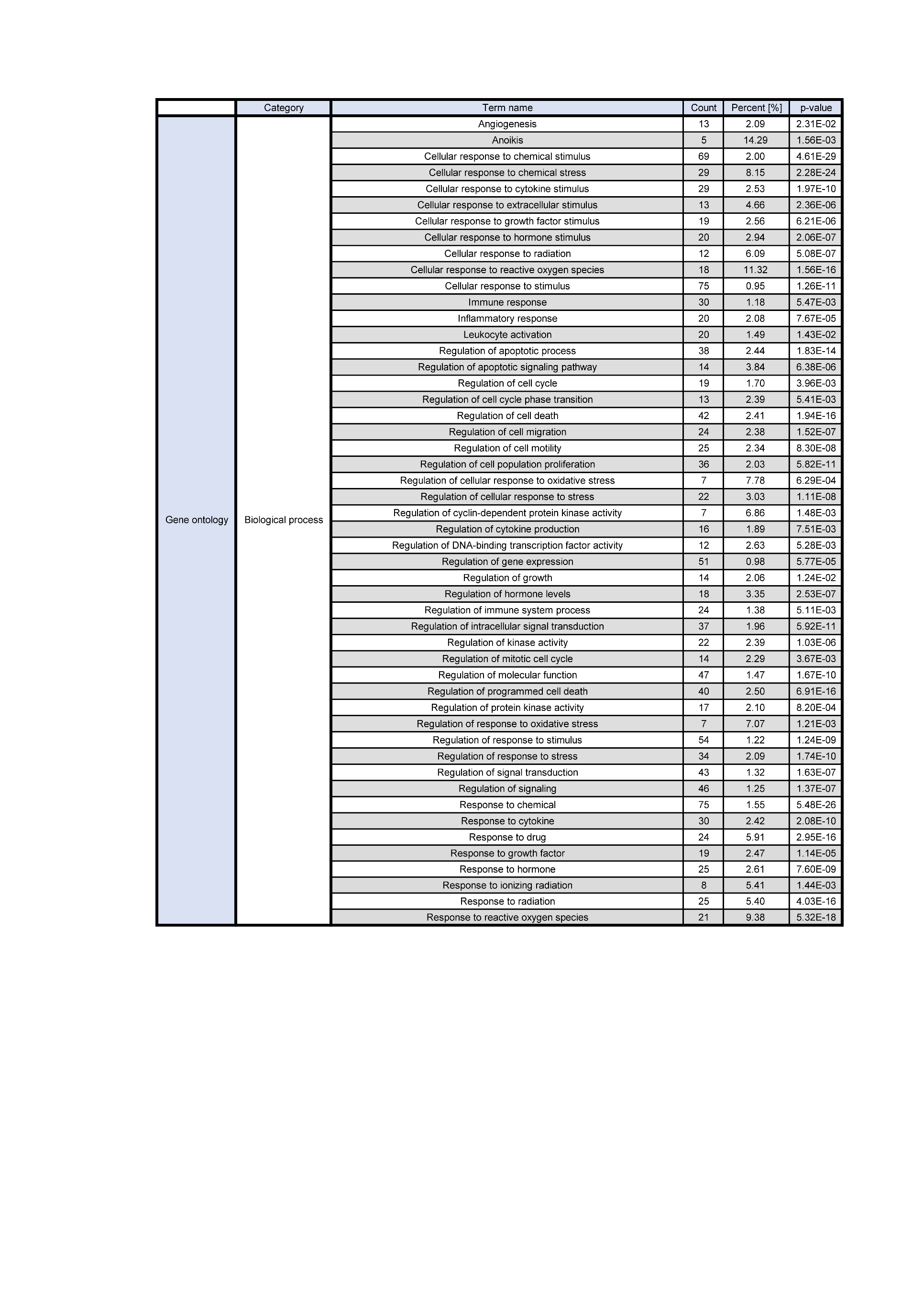


**
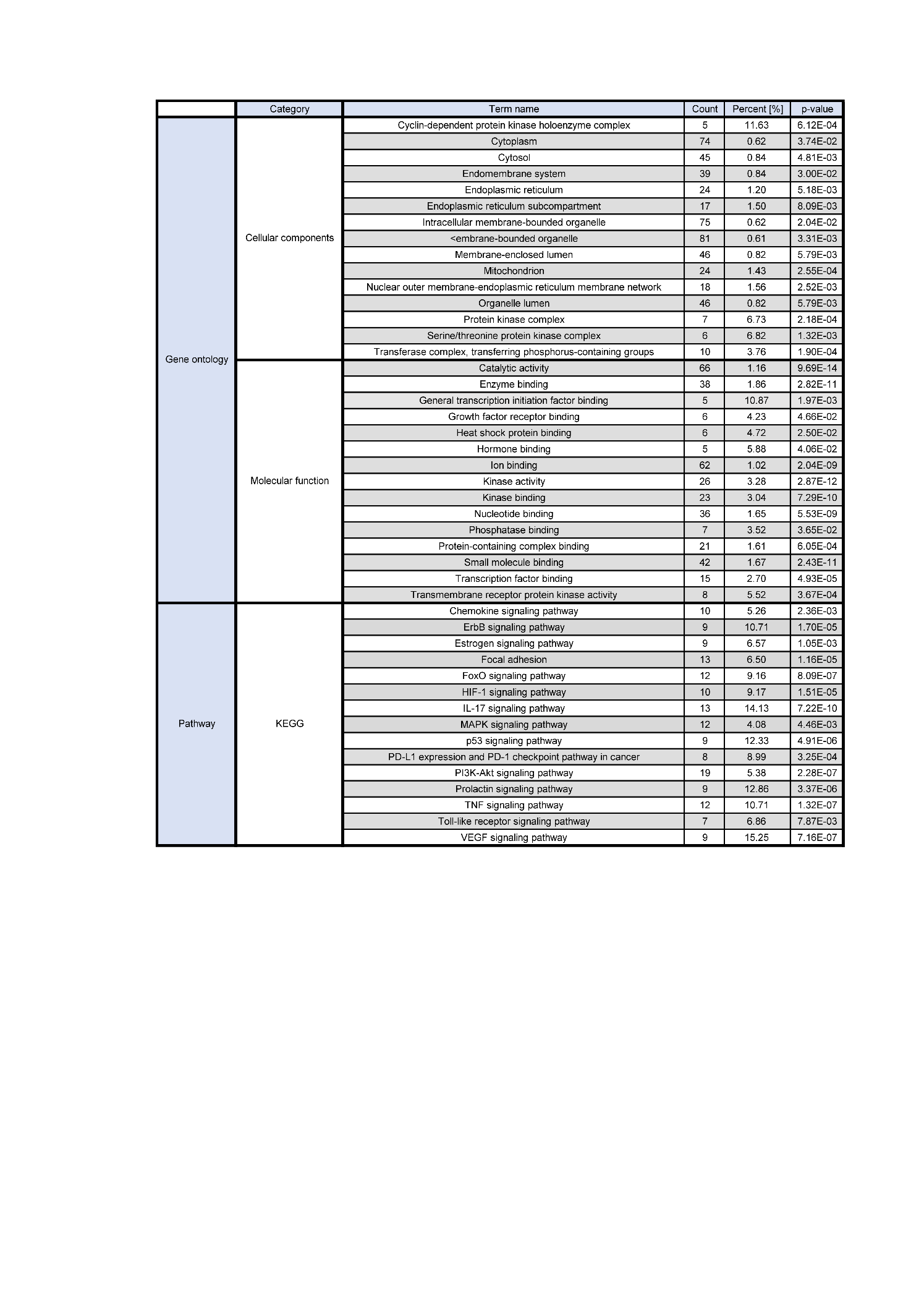
**

**Supplementary Figure S2. Gene ontology and pathway enrichment analysis for the liver cancer-related targets of FDY003.**


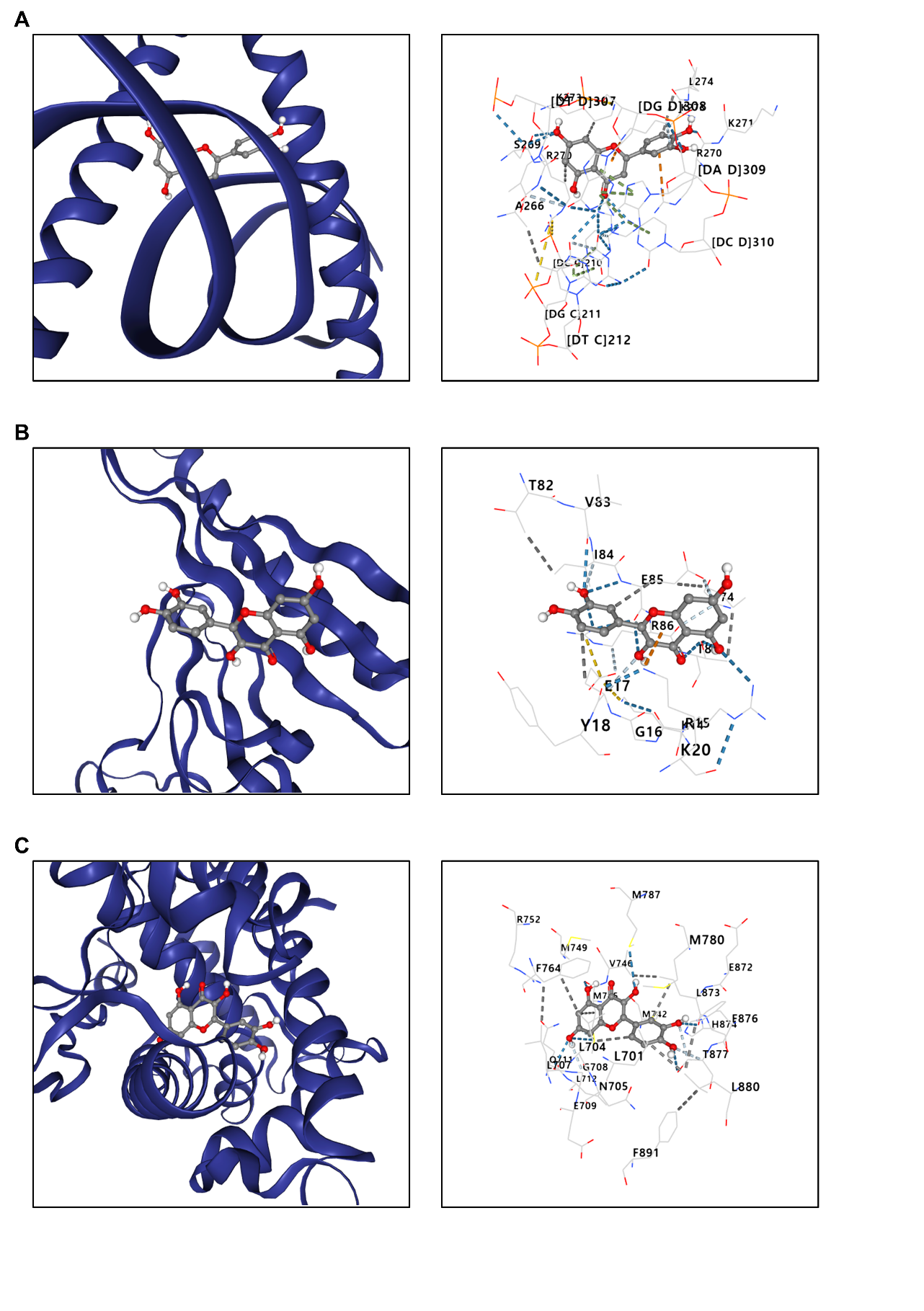


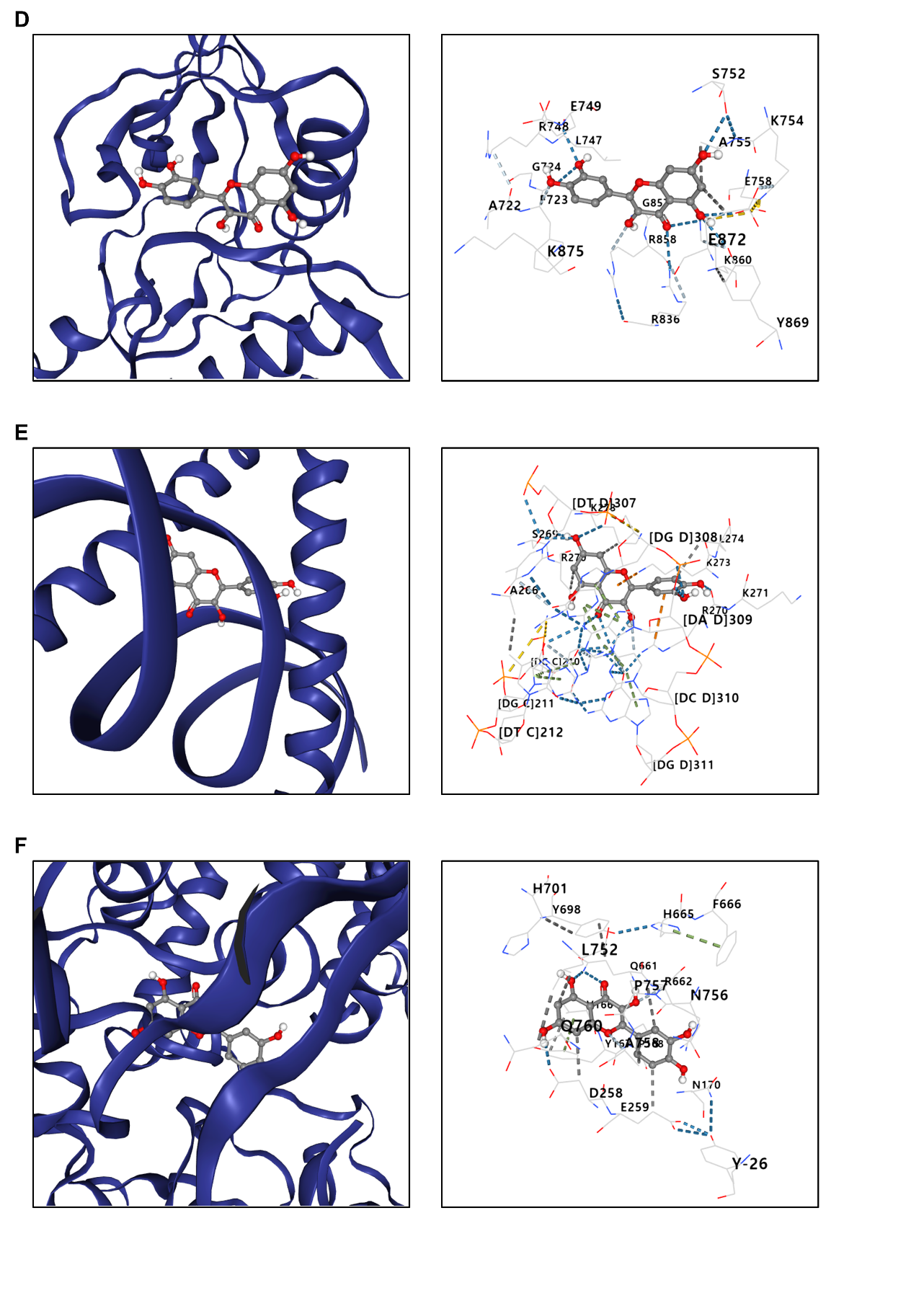


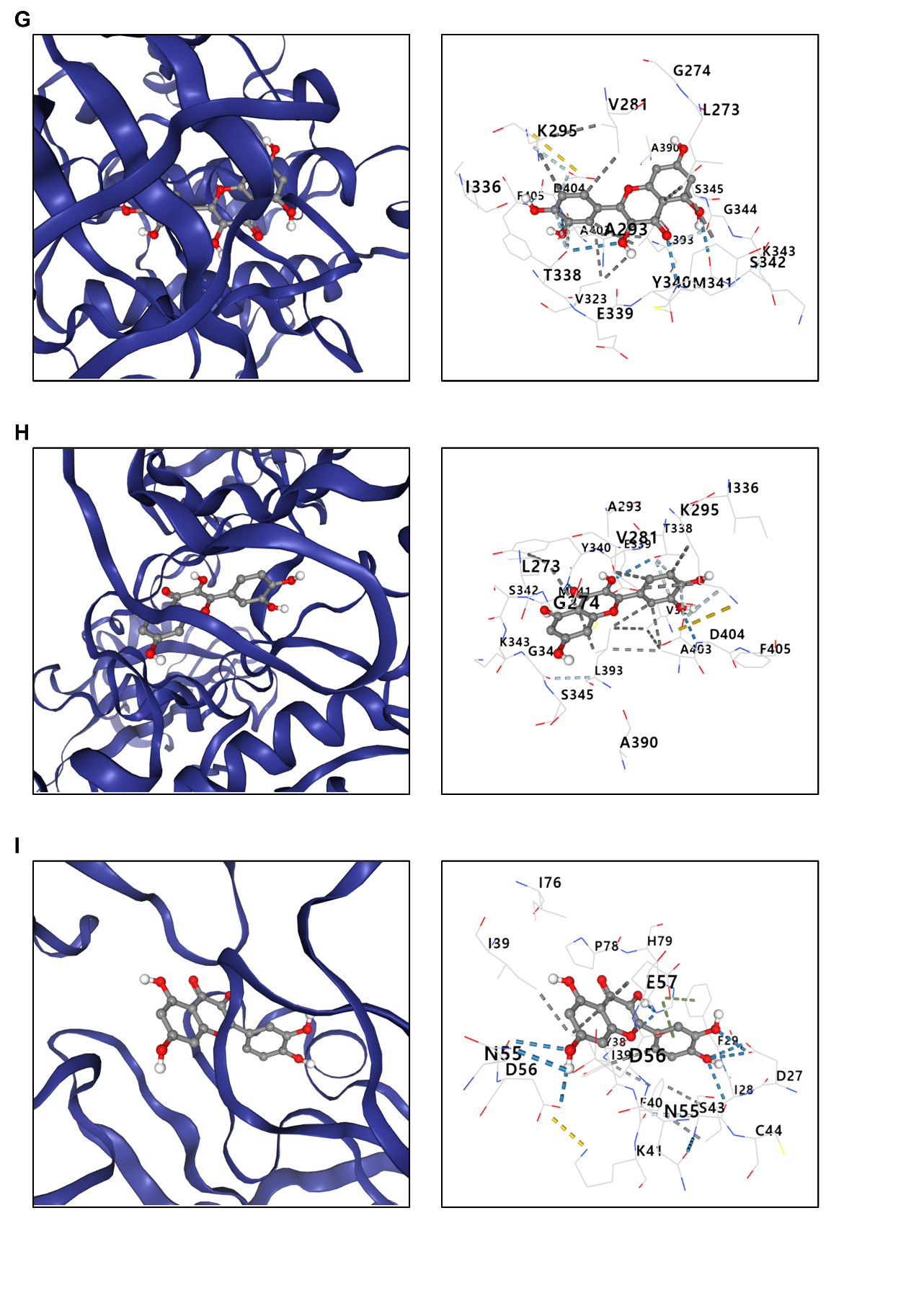


**Supplementary Figure S3. Analysis of binding affinities between the bioactive chemical components of FDY003 and their targets. (A)** Luteolin–JUN (binding energy = −8.8 kcal/mol). **(B)** Quercetin–AKT1 (binding energy = −6.6). **(C)** Quercetin–AR (binding energy = −8.9 kcal/mol). **(D)** Quercetin–EGFR (binding energy = −8.2 kcal/mol). **(E)** Quercetin–JUN (binding energy = −9.2 kcal/mol). **(F)** Quercetin–PIK3R1 (binding energy = −9.7 kcal/mol). **(G)** Quercetin–SRC (binding energy = −8.7 kcal/mol). **(H)** Quercetin–TP53 (binding energy = −8.7 kcal/mol). **(I)** Quercetin–VEGFA (binding energy = −8.3 kcal/mol).

## Supplementary Tables

**Supplementary Table S1. Clinical information of the included patients for the survival analysis.**

| Pathology | |
| --- | --- |
| Stage | All |
| Grade | All |
| AJCC_T | All |
| Vascular invasion | All |
| Patient | |
| Gender | All |
| Race | All |
| Sorafenib treatment | All |
| Risk factors | |
| Alcohol consumption | All |
| Hepatitis virus | All |
| Mutation burden | All |

**Supplementary Table S2. List of chemical components of FDY003.**

| Herbal medicines | Chemical components | OB | Caco-2 | DL | Hdon | Hacc | MW | AlogP | RBN |
| --- | --- | --- | --- | --- | --- | --- | --- | --- | --- |
| LjT | (-)-(3R,8S,9R,9aS,10aS)-9-ethenyl-8-(beta-D-glucopyranosyloxy)-2,3,9,9a,10,10a-hexahydro-5-oxo-5H,8H-pyrano[4,3-d]oxazolo[3,2-a]pyridine-3-carboxylic acid | 3.22 | -1.72 | 0.80 | 5 | 12 | 443.45 | -2.71 | 5 |
| LjT | (-)-(3R,8S,9R,9aS,10aS)-9-ethenyl-8-(beta-D-glucopyranosyloxy)-2,3,9,9a,10,10a-hexahydro-5-oxo-5H,8H-pyrano[4,3-d]oxazolo[3,2-a]pyridine-3-carboxylic acid_qt | 87.47 | -0.55 | 0.23 | 2 | 7 | 281.29 | -0.96 | 2 |
| LjT | (-)-Caryophyllene oxide | 32.67 | 1.58 | 0.13 | 0 | 1 | 220.39 | 3.52 | 0 |
| LjT | ()-Menthol | 59.33 | 1.27 | 0.03 | 1 | 1 | 156.30 | 2.78 | 1 |
| LjT | (-)-α-Pinene | 46.25 | 1.85 | 0.05 | 0 | 0 | 136.26 | 2.87 | 0 |
| LjT | ()-α-Terpineol | 46.30 | 1.28 | 0.03 | 1 | 1 | 154.28 | 2.42 | 1 |
| LjT | (+)-Ledol | 16.96 | 1.43 | 0.12 | 1 | 1 | 222.41 | 3.20 | 0 |
| LjT | (1alpha,3R,4alpha,5R)-3,4,5-Tris[[(2E)-3-(3,4-dihydroxyphenyl)-1-oxo-2-propen-1-yl]oxy]-1-hydroxycyclohexanecarboxylic acid | 3.01 | -1.46 | 0.45 | 7 | 15 | 677.63 | 2.86 | 13 |
| LjT | (1R,2R,4R)-Dihydrocarveol | 51.17 | 1.32 | 0.03 | 1 | 1 | 154.28 | 2.58 | 1 |
| LjT | (1R,4S,4aR)-1-isopropyl-4-methyl-7-methylene-2,3,4,4a,5,6-hexahydro-1H-naphthalene | 19.50 | 1.84 | 0.08 | 0 | 0 | 204.39 | 4.80 | 1 |
| LjT | (1S,2S)-2-isopropenyl-4-isopropylidene-1-methyl-1-vinylcyclohexane | 34.47 | 1.87 | 0.06 | 0 | 0 | 204.39 | 4.93 | 2 |
| LjT | (1S,4E,8E,10R)-4,8,11,11-tetramethylbicyclo[8.1.0]undeca-4,8-diene | 21.69 | 1.86 | 0.08 | 0 | 0 | 204.39 | 4.70 | 0 |
| LjT | (2S)-2-methylbutan-1-ol | 81.23 | 1.06 | 0.00 | 1 | 1 | 88.17 | 1.29 | 2 |
| LjT | (3R,4aS,5R,6R)-6-hydroxy-3-methoxy-5-vinyl-4,4a,5,6-tetrahydro-3H-pyrano[5,4-c]pyran-1-one | 60.89 | 0.09 | 0.10 | 1 | 5 | 226.25 | -0.04 | 2 |
| LjT | (5Z,9Z)-6,10,14-trimethylpentadeca-5,9,13-trien-2-one | 37.84 | 1.58 | 0.10 | 0 | 1 | 262.48 | 5.45 | 9 |
| LjT | (E,E,E)-3,7-11,16-tetramethyl hexadeca-2,6,10,14-tetraen-1-ol | 42.48 | 1.42 | 0.14 | 1 | 1 | 290.54 | 6.60 | 11 |
| LjT | (E,Z)-farnesol | 36.73 | 1.32 | 0.06 | 1 | 1 | 222.41 | 4.76 | 7 |
| LjT | (S)-phenethyl 2-bromopropanoate | 9.10 | 1.27 | 0.05 | 0 | 2 | 257.14 | 3.19 | 5 |
| LjT | (Z,E)-farnesol | 41.14 | 1.34 | 0.06 | 1 | 1 | 222.41 | 4.76 | 6 |
| LjT | [(1S)-endo]-(-)-Borneol | 83.54 | 1.22 | 0.05 | 1 | 1 | 154.28 | 1.98 | 0 |
| LjT | 1,6-Dicyclohexylhexane | 15.40 | 1.81 | 0.11 | 0 | 0 | 250.52 | 7.43 | 7 |
| LjT | 1H,3H-Pyrano(3,4-c)pyran-1-one, 5-ethenyl-6-(beta-D-glucopyranosyloxy)-4,4a,5,6-tetrahydro-, (4aS-(4aalpha,5beta,6alpha))- | 4.96 | -1.08 | 0.38 | 4 | 9 | 358.38 | -1.59 | 4 |
| LjT | 2-(2,4-dimethoxyphenyl)-3-hydroxy-7-methoxy-chromone | 12.94 | 0.72 | 0.33 | 1 | 6 | 328.34 | 2.52 | 4 |
| LjT | 2-(3,4-dimethoxyphenyl)-5-hydroxy-7-methoxy-chromone | 29.24 | 0.90 | 0.34 | 1 | 6 | 328.34 | 2.82 | 3 |
| LjT | 2,3-DIMETHYLPYRAZINE | 30.82 | 1.07 | 0.02 | 0 | 2 | 108.16 | 0.09 | 0 |
| LjT | 2-[(1R,3S,4S)-3-isopropenyl-4-methyl-4-vinylcyclohexyl]propan-2-ol | 19.03 | 1.37 | 0.07 | 1 | 1 | 222.41 | 3.70 | 3 |
| LjT | 2-[(2S,5R)-5-ethenyl-5-methyloxolan-2-yl]propan-2-ol | 68.08 | 0.99 | 0.04 | 1 | 2 | 170.28 | 1.43 | 2 |
| LjT | 2-bromododecane | 17.60 | 1.82 | 0.03 | 0 | 0 | 249.27 | 5.89 | 9 |
| LjT | 2H-Pyran-5-carboxylic acid, 4-(2,2-dimethoxyethyl)-3-ethenyl-2-(beta-D-glucopyranosyloxy)-3,4-dihydro-, methyl ester, (2S,3R,4S)- | 22.59 | -1.25 | 0.45 | 4 | 11 | 434.49 | -1.73 | 10 |
| LjT | 2-isopropenyl-5-methylhex-4-enal | 27.69 | 1.33 | 0.02 | 0 | 1 | 152.26 | 2.83 | 4 |
| LjT | 2-methylpentadecane | 4.35 | 1.82 | 0.06 | 0 | 0 | 226.50 | 7.47 | 12 |
| LjT | 3,4-Dicaffeoylquinic acid | 1.71 | -1.40 | 0.69 | 6 | 12 | 515.48 | 0.88 | 9 |
| LjT | 3,4-Dimethyl-2-hexanone | 35.01 | 1.38 | 0.01 | 0 | 1 | 128.24 | 2.05 | 3 |
| LjT | 3,4-di-O-caffeoylquinic acid methyl ester | 1.71 | -1.02 | 0.69 | 6 | 12 | 530.52 | 1.81 | 10 |
| LjT | 3,5-di-O-caffeoylquinic acid methyl ester | 1.73 | -0.96 | 0.68 | 6 | 12 | 530.52 | 1.81 | 10 |
| LjT | 3-Hexenol | 62.74 | 1.05 | 0.01 | 1 | 1 | 100.18 | 1.44 | 3 |
| LjT | 3-Methyl-2-pent-2-enyl-cyclopent-2-enone | 25.28 | 1.52 | 0.03 | 0 | 1 | 164.27 | 2.97 | 3 |
| LjT | 3-O-Methylquercetin | 10.10 | 0.20 | 0.30 | 4 | 7 | 316.28 | 1.57 | 2 |
| LjT | 4,5-Dicaffeoylquinic acid | 1.73 | -1.37 | 0.69 | 6 | 12 | 515.48 | 0.88 | 9 |
| LjT | 4,5-di-O-caffeoylquinic acid methyl ester | 1.73 | -0.99 | 0.69 | 6 | 12 | 530.52 | 1.81 | 10 |
| LjT | 4,5'-Retro-.beta.,.beta.-Carotene-3,3'-dione, 4',5'-didehydro- | 31.22 | 1.17 | 0.55 | 0 | 2 | 562.90 | 9.27 | 9 |
| LjT | 4-caffeoylquinic acid | 10.48 | -1.52 | 0.33 | 5 | 9 | 353.33 | -1.09 | 5 |
| LjT | 4-stearylmorpholine | 14.80 | 1.48 | 0.27 | 0 | 2 | 339.68 | 7.72 | 17 |
| LjT | 5-O-Caffeoyl quinic acid butyl ester | 8.77 | -0.65 | 0.41 | 5 | 9 | 410.46 | 1.16 | 9 |
| LjT | 7-epi-Loganin | 4.78 | -1.65 | 0.44 | 5 | 10 | 390.43 | -2.08 | 5 |
| LjT | 7-epi-Loganin_qt | 85.12 | -0.28 | 0.10 | 2 | 5 | 228.27 | -0.33 | 2 |
| LjT | 7-epi-Vogeloside | 46.13 | -1.30 | 0.58 | 4 | 11 | 432.47 | -0.19 | 6 |
| LjT | 7-epi-Vogeloside_qt | 33.26 | 0.04 | 0.16 | 1 | 6 | 270.31 | 0.99 | 3 |
| LjT | 8-epiloganin | 11.68 | -1.74 | 0.44 | 5 | 10 | 390.43 | -2.08 | 5 |
| LjT | 8-epiloganin_qt | 26.42 | -0.64 | 0.10 | 2 | 5 | 228.27 | -0.33 | 2 |
| LjT | 9-epi-(E)-caryophyllene | 30.28 | 1.83 | 0.09 | 0 | 0 | 204.39 | 4.75 | 0 |
| LjT | Akebiasaponin D | 1.67 | -3.02 | 0.07 | 11 | 18 | 929.23 | 0.44 | 10 |
| LjT | Akebiasaponin D_qt | 16.44 | 0.19 | 0.74 | 3 | 4 | 472.78 | 5.33 | 2 |
| LjT | Alloaromadedrene | 53.46 | 1.83 | 0.10 | 0 | 0 | 204.39 | 4.22 | 0 |
| LjT | Amylol | 76.16 | 1.02 | 0.00 | 1 | 1 | 88.17 | 1.43 | 3 |
| LjT | Apigenin | 23.06 | 0.43 | 0.21 | 3 | 5 | 270.25 | 2.33 | 1 |
| LjT | Astragalin | 14.03 | -1.34 | 0.74 | 7 | 11 | 448.41 | -0.32 | 4 |
| LjT | Atractylodin | 44.49 | 2.00 | 0.05 | 0 | 1 | 182.23 | 3.83 | 1 |
| LjT | BNL | 41.30 | 1.79 | 0.04 | 0 | 0 | 166.34 | 4.69 | 1 |
| LjT | BZM | 18.64 | 1.36 | 0.09 | 0 | 2 | 212.26 | 3.27 | 4 |
| LjT | C09704 | 29.56 | 1.37 | 0.06 | 1 | 1 | 222.41 | 4.56 | 7 |
| LjT | Caeruloside C | 55.64 | -2.91 | 0.73 | 7 | 15 | 550.57 | -3.70 | 11 |
| LjT | Caeruloside C_qt | 5.40 | -1.64 | 0.37 | 4 | 10 | 388.41 | -1.95 | 8 |
| LjT | Caffeate | 54.97 | 0.27 | 0.05 | 3 | 4 | 180.17 | 1.37 | 2 |
| LjT | CAM | 67.17 | 1.29 | 0.05 | 0 | 1 | 152.26 | 1.94 | 0 |
| LjT | Caprylic acid | 16.40 | 0.90 | 0.02 | 1 | 2 | 144.24 | 2.72 | 6 |
| LjT | Cedrol | 16.23 | 1.35 | 0.12 | 1 | 1 | 222.41 | 3.16 | 0 |
| LjT | Centauroside | 4.37 | -2.95 | 0.43 | 8 | 19 | 758.80 | -3.11 | 16 |
| LjT | Centauroside_qt | 55.79 | -0.84 | 0.50 | 2 | 9 | 434.48 | 0.38 | 10 |
| LjT | Chlorogenic acid | 11.93 | -1.03 | 0.33 | 6 | 9 | 354.34 | -0.42 | 5 |
| LjT | Chrysoeriol | 35.85 | 0.39 | 0.27 | 3 | 6 | 300.28 | 2.32 | 2 |
| LjT | CIS-2-PENTENOL | 66.40 | 1.02 | 0.00 | 1 | 1 | 86.15 | 1.12 | 2 |
| LjT | Copaene | 29.47 | 1.81 | 0.12 | 0 | 0 | 204.39 | 4.17 | 1 |
| LjT | Corymbosin | 51.96 | 0.88 | 0.41 | 1 | 7 | 358.37 | 2.80 | 5 |
| LjT | Cosmetin | 9.68 | -1.08 | 0.74 | 6 | 10 | 432.41 | 0.43 | 4 |
| LjT | D-Camphene | 34.98 | 1.81 | 0.04 | 0 | 0 | 136.26 | 2.93 | 0 |
| LjT | Dehydroxymorroniside | 20.69 | -1.25 | 0.46 | 4 | 10 | 388.41 | -2.25 | 5 |
| LjT | Dehydroxymorroniside_qt | 29.40 | 0.12 | 0.10 | 1 | 5 | 226.25 | -0.51 | 2 |
| LjT | Desaspidinol-A | 30.51 | 0.64 | 0.05 | 2 | 4 | 182.19 | 1.02 | 2 |
| LjT | Dinethylsecologanoside | 48.46 | -1.37 | 0.48 | 4 | 12 | 434.44 | -0.79 | 10 |
| LjT | Dinethylsecologanoside_qt | 4.50 | -0.21 | 0.12 | 1 | 7 | 272.28 | 0.39 | 7 |
| LjT | Disacoside B | 1.99 | -3.95 | 0.03 | 13 | 22 | 1075.39 | -0.41 | 12 |
| LjT | Disacoside B_qt | 15.83 | 0.17 | 0.74 | 3 | 4 | 472.78 | 5.33 | 2 |
| LjT | Dodekan | 17.74 | 1.79 | 0.02 | 0 | 0 | 170.38 | 5.85 | 9 |
| LjT | Eriodictyol-7-o-glucoside | 17.57 | -1.34 | 0.78 | 7 | 11 | 450.43 | 0.13 | 4 |
| LjT | Eriodyctiol (flavanone) | 41.35 | 0.05 | 0.24 | 4 | 6 | 288.27 | 2.03 | 1 |
| LjT | ETHYL FURAN | 55.07 | 1.68 | 0.01 | 0 | 1 | 96.14 | 1.75 | 1 |
| LjT | Ethyl linolenate | 46.10 | 1.54 | 0.20 | 0 | 2 | 306.54 | 6.55 | 15 |
| LjT | Ethylpalmitate | 18.99 | 1.41 | 0.14 | 0 | 2 | 284.54 | 6.97 | 16 |
| LjT | Eudesmol | 35.38 | 1.28 | 0.09 | 1 | 1 | 224.43 | 4.10 | 1 |
| LjT | Eugenol | 56.24 | 1.35 | 0.04 | 1 | 2 | 164.22 | 2.55 | 3 |
| LjT | Farnesene | 17.42 | 1.95 | 0.05 | 0 | 0 | 204.39 | 5.52 | 7 |
| LjT | Farnesol | 28.44 | 1.32 | 0.06 | 1 | 1 | 222.41 | 4.76 | 7 |
| LjT | Farnesol acetate | 21.97 | 1.44 | 0.11 | 0 | 2 | 264.45 | 5.14 | 9 |
| LjT | Flavone der. | 27.12 | 0.83 | 0.27 | 1 | 5 | 298.31 | 2.84 | 3 |
| LjT | Fulvotomentoside A | 3.30 | -4.60 | 0.02 | 15 | 26 | 1207.52 | -1.65 | 14 |
| LjT | Geraniol | 23.93 | 1.19 | 0.02 | 1 | 1 | 154.28 | 2.93 | 4 |
| LjT | Germacrene D | 19.22 | 1.83 | 0.06 | 0 | 0 | 204.39 | 5.14 | 1 |
| LjT | Ginnol | 11.33 | 1.46 | 0.43 | 1 | 1 | 424.89 | 12.37 | 26 |
| LjT | GLO | 24.44 | -1.93 | 0.03 | 5 | 6 | 180.18 | -2.68 | 5 |
| LjT | Guaiol | 38.77 | 1.36 | 0.09 | 1 | 1 | 222.41 | 3.91 | 1 |
| LjT | Hederagenol | 22.42 | 0.10 | 0.74 | 3 | 4 | 472.78 | 5.33 | 2 |
| LjT | Helixin | 9.63 | -1.81 | 0.14 | 7 | 12 | 751.07 | 3.24 | 6 |
| LjT | HEPTACOSANE | 8.18 | 1.88 | 0.36 | 0 | 0 | 380.83 | 12.69 | 24 |
| LjT | Heptadecyloxirane | 12.44 | 1.67 | 0.15 | 0 | 1 | 282.57 | 7.61 | 16 |
| LjT | Heptenal | 37.16 | 1.30 | 0.01 | 0 | 1 | 112.19 | 2.29 | 4 |
| LjT | Hexadienal | 38.55 | 1.25 | 0.01 | 0 | 1 | 96.14 | 1.39 | 2 |
| LjT | Hexanal | 55.71 | 1.25 | 0.01 | 0 | 1 | 100.18 | 1.85 | 4 |
| LjT | Hexenal | 46.01 | 1.29 | 0.01 | 0 | 1 | 98.16 | 1.83 | 3 |
| LjT | Hexene | 60.01 | 1.78 | 0.00 | 0 | 0 | 84.18 | 2.72 | 3 |
| LjT | Hyacinthin | 38.65 | 1.31 | 0.02 | 0 | 1 | 120.16 | 1.52 | 2 |
| LjT | Hydnocarpin | 2.06 | -0.13 | 0.94 | 4 | 9 | 464.45 | 3.21 | 4 |
| LjT | Hydroquinone | 29.26 | 0.89 | 0.02 | 2 | 2 | 110.12 | 1.30 | 0 |
| LjT | HYKOP | 32.79 | 0.25 | 0.05 | 3 | 4 | 182.19 | 1.39 | 3 |
| LjT | Hyperin | 6.94 | -1.42 | 0.77 | 8 | 12 | 464.41 | -0.59 | 4 |
| LjT | Indole | 34.38 | 1.81 | 0.03 | 1 | 0 | 117.16 | 2.12 | 0 |
| LjT | Inositol | 18.86 | -1.52 | 0.05 | 6 | 6 | 180.18 | -3.06 | 0 |
| LjT | Loniceracetalides B | 5.48 | -1.44 | 0.63 | 4 | 12 | 476.53 | -0.17 | 9 |
| LjT | Loniceracetalides B_qt | 61.19 | -0.09 | 0.19 | 1 | 7 | 314.37 | 1.00 | 6 |
| LjT | Isobutyl tiglate | 24.51 | 1.30 | 0.02 | 0 | 2 | 156.25 | 2.76 | 4 |
| LjT | Isochlorogenic acid C | 1.78 | -1.32 | 0.69 | 7 | 12 | 516.49 | 1.56 | 9 |
| LjT | Isochlorogenic,acid | 1.79 | -1.10 | 0.69 | 7 | 12 | 516.49 | 1.56 | 9 |
| LjT | Isothiazole, trimethyl- | 67.44 | 1.42 | 0.02 | 0 | 1 | 127.23 | 1.76 | 0 |
| LjT | Junipene | 44.07 | 1.82 | 0.11 | 0 | 0 | 204.39 | 4.18 | 0 |
| LjT | Kaempferol | 41.88 | 0.26 | 0.24 | 4 | 6 | 286.25 | 1.77 | 1 |
| LjT | Kryptoxanthin | 47.25 | 1.69 | 0.57 | 1 | 1 | 552.96 | 10.76 | 10 |
| LjT | Lauric acid | 23.59 | 1.02 | 0.04 | 1 | 2 | 200.36 | 4.54 | 10 |
| LjT | L-Bornyl acetate | 65.52 | 1.29 | 0.08 | 0 | 2 | 196.32 | 2.35 | 2 |
| LjT | Leukol | 35.25 | 1.59 | 0.03 | 0 | 1 | 129.17 | 2.02 | 0 |
| LjT | Lignoceric acid | 14.90 | 1.24 | 0.33 | 1 | 2 | 368.72 | 10.02 | 22 |
| LjT | Linalool | 49.37 | 0.86 | 0.04 | 1 | 2 | 170.28 | 1.43 | 4 |
| LjT | LINALOOL (D) | 38.29 | 1.29 | 0.02 | 1 | 1 | 154.28 | 2.74 | 4 |
| LjT | Loganic | 34.96 | -0.58 | 0.05 | 4 | 5 | 176.19 | -0.59 | 1 |
| LjT | Loganic acid | 4.92 | -1.81 | 0.40 | 6 | 10 | 376.40 | -2.33 | 4 |
| LjT | Loganic acid_qt | 114.65 | -0.59 | 0.09 | 3 | 5 | 214.24 | -0.58 | 1 |
| LjT | Loganin | 5.90 | -1.48 | 0.44 | 5 | 10 | 390.43 | -2.08 | 5 |
| LjT | Loniceracetalide A | 28.29 | -1.31 | 0.58 | 4 | 11 | 460.53 | -1.16 | 8 |
| LjT | Loniceracetalide A_qt | 89.38 | -0.30 | 0.17 | 1 | 6 | 298.37 | 0.59 | 5 |
| LjT | Loniceracetalide B | 10.77 | -1.37 | 0.58 | 4 | 11 | 460.53 | -1.16 | 8 |
| LjT | Loniceracetalide B_qt | 89.28 | 0.16 | 0.17 | 1 | 6 | 298.37 | 0.59 | 5 |
| LjT | Luteolin | 36.16 | 0.19 | 0.25 | 4 | 6 | 286.25 | 2.07 | 1 |
| LjT | Luteolin-7-o-glucoside | 7.29 | -1.23 | 0.78 | 7 | 11 | 448.41 | 0.16 | 4 |
| LjT | Macranthoidin A | 4.15 | -4.97 | 0.01 | 16 | 27 | 1237.55 | -2.16 | 15 |
| LjT | Macranthoidin B | 6.69 | -5.80 | 0.01 | 19 | 32 | 1399.71 | -3.91 | 18 |
| LjT | Madreselvin A | 6.03 | -2.50 | 0.59 | 10 | 17 | 640.60 | -2.08 | 8 |
| LjT | Madreselvin B | 3.01 | -2.42 | 0.26 | 12 | 20 | 788.72 | -0.36 | 11 |
| LjT | Mandenol | 42.00 | 1.46 | 0.19 | 0 | 2 | 308.56 | 6.99 | 16 |
| LjT | Methional | 53.62 | 1.04 | 0.00 | 0 | 1 | 104.19 | 0.55 | 3 |
| LjT | methyl (1R,4aS,6S,7R,7aS)-1,6-dihydroxy-7-methyl-1,4a,5,6,7,7a-hexahydrocyclopenta[d]pyran-4-carboxylate | 29.99 | -0.36 | 0.10 | 2 | 5 | 228.27 | -0.33 | 2 |
| LjT | Methyl caffeate | 30.68 | 0.54 | 0.06 | 2 | 4 | 194.20 | 1.62 | 3 |
| LjT | Methyl chlorogenate | 9.97 | -0.74 | 0.36 | 5 | 9 | 368.37 | -0.16 | 6 |
| LjT | Methyl isomyristate | 20.26 | 1.38 | 0.08 | 0 | 2 | 242.45 | 5.50 | 12 |
| LjT | METHYL LINOLEATE | 41.93 | 1.44 | 0.17 | 0 | 2 | 294.53 | 6.64 | 15 |
| LjT | Methyl myristate | 19.68 | 1.36 | 0.08 | 0 | 2 | 242.45 | 5.71 | 13 |
| LjT | Methyl octadeca-8,11-dienoate | 41.93 | 1.46 | 0.17 | 0 | 2 | 294.53 | 6.64 | 15 |
| LjT | Methyl palmitate | 18.09 | 1.37 | 0.12 | 0 | 2 | 270.51 | 6.62 | 15 |
| LjT | Methyl-9-methyl tetradecanoate | 13.70 | 1.35 | 0.09 | 0 | 2 | 256.48 | 5.96 | 13 |
| LjT | Methyllinolenate | 46.15 | 1.48 | 0.17 | 0 | 2 | 292.51 | 6.20 | 14 |
| LjT | Methyl-p-coumarate | 20.14 | 0.83 | 0.05 | 1 | 3 | 178.20 | 1.89 | 3 |
| LjT | Muurolene | 19.50 | 1.84 | 0.08 | 0 | 0 | 204.39 | 4.75 | 1 |
| LjT | Myristic acid | 21.18 | 1.07 | 0.07 | 1 | 2 | 228.42 | 5.46 | 12 |
| LjT | Neochlorogenic acid | 10.65 | -1.43 | 0.33 | 5 | 9 | 353.33 | -1.09 | 5 |
| LjT | Nerol | 35.66 | 1.15 | 0.02 | 1 | 1 | 154.28 | 2.93 | 4 |
| LjT | Neryl acetate | 57.47 | 1.25 | 0.04 | 0 | 2 | 196.32 | 3.31 | 6 |
| LjT | NON | 26.74 | 0.96 | 0.03 | 1 | 2 | 172.30 | 3.63 | 8 |
| LjT | Nonacosanol | 10.57 | 1.48 | 0.43 | 1 | 1 | 424.89 | 12.38 | 27 |
| LjT | Nonanal | 40.28 | 1.31 | 0.02 | 0 | 1 | 142.27 | 3.22 | 7 |
| LjT | Nonanoic acid | 40.51 | 0.92 | 0.02 | 1 | 2 | 158.27 | 3.17 | 7 |
| LjT | Ochnaflavone | 2.54 | -0.11 | 0.57 | 5 | 10 | 538.48 | 4.67 | 4 |
| LjT | Octadecylglycol | 16.18 | 1.07 | 0.19 | 1 | 2 | 314.62 | 7.23 | 19 |
| LjT | Octanol | 21.06 | 1.16 | 0.01 | 1 | 1 | 130.26 | 2.80 | 6 |
| LjT | Olean-12-en-28-oic acid, 23-hydroxy-3-((O-beta-D-xylopyranosyl-(1-3)-O-6-deoxy-alpha-L-mannopyranosyl-(1-2)-alpha-L-arabinopyranosyl)oxy)-, (3beta,4alpha)- | 3.74 | -2.34 | 0.06 | 9 | 16 | 883.20 | 2.00 | 8 |
| LjT | Oleanolic acid | 29.02 | 0.59 | 0.76 | 2 | 3 | 456.78 | 6.42 | 1 |
| LjT | o-Thymol | 43.28 | 1.58 | 0.03 | 1 | 1 | 150.24 | 3.24 | 1 |
| LjT | p-Coumaric acid | 43.29 | 0.46 | 0.04 | 2 | 3 | 164.17 | 1.64 | 2 |
| LjT | PEL | 44.03 | 1.11 | 0.02 | 1 | 1 | 122.18 | 1.55 | 2 |
| LjT | Pent-3-en-2-one | 50.20 | 1.25 | 0.00 | 0 | 1 | 84.13 | 0.86 | 1 |
| LjT | Pentadecene | 17.72 | 1.84 | 0.05 | 0 | 0 | 210.45 | 6.82 | 12 |
| LjT | PHB | 30.15 | 0.39 | 0.03 | 2 | 3 | 138.13 | 1.17 | 1 |
| LjT | PHYTANTRIOL | 23.20 | 0.45 | 0.18 | 3 | 3 | 330.62 | 5.47 | 14 |
| LjT | Phytofluene | 43.18 | 2.29 | 0.50 | 0 | 0 | 543.02 | 14.10 | 19 |
| LjT | Prenal | 48.87 | 1.23 | 0.00 | 0 | 1 | 84.13 | 1.36 | 1 |
| LjT | Propyl vinyl ketone | 72.27 | 1.35 | 0.01 | 0 | 1 | 98.16 | 1.58 | 3 |
| LjT | Protocatechuic acid | 25.37 | 0.10 | 0.04 | 3 | 4 | 154.13 | 0.90 | 1 |
| LjT | PTL | 59.53 | 1.21 | 0.00 | 0 | 1 | 86.15 | 1.40 | 3 |
| LjT | Quercetin-3-o-β-D-glu | 1.81 | -1.40 | 0.79 | 8 | 13 | 478.39 | -0.38 | 4 |
| LjT | Quinic acid | 55.92 | -1.79 | 0.06 | 4 | 6 | 191.18 | -3.07 | 1 |
| LjT | Rhoifolin | 6.68 | -1.87 | 0.77 | 8 | 14 | 578.57 | -0.43 | 6 |
| LjT | SCG | 23.59 | -1.58 | 0.36 | 4 | 10 | 388.41 | -1.98 | 8 |
| LjT | Scolymoside | 3.84 | -2.16 | 0.73 | 9 | 15 | 594.57 | -0.70 | 6 |
| LjT | Secologanate | 17.56 | -1.90 | 0.33 | 5 | 10 | 374.38 | -2.23 | 7 |
| LjT | Secologanic acid_qt | 73.14 | -0.51 | 0.07 | 2 | 5 | 212.22 | -0.49 | 4 |
| LjT | Secologanic dibutylacetal | 20.05 | -0.97 | 0.67 | 4 | 11 | 546.73 | 1.84 | 18 |
| LjT | Secologanic dibutylacetal_qt | 53.65 | 0.34 | 0.29 | 1 | 6 | 384.57 | 3.58 | 15 |
| LjT | Secologanin dimethylacetal_qt | 2.71 | -0.07 | 0.11 | 1 | 6 | 272.33 | 0.01 | 7 |
| LjT | Secologanin_qt | 33.33 | -0.10 | 0.08 | 1 | 5 | 226.25 | -0.23 | 5 |
| LjT | Secologanoside | 26.92 | -2.31 | 0.37 | 4 | 11 | 388.36 | -3.64 | 7 |
| LjT | Secologanoside 7-methylester | 3.88 | -1.66 | 0.45 | 5 | 12 | 420.41 | -1.04 | 9 |
| LjT | Secologanoside 7-methylester_qt | 38.01 | -0.37 | 0.11 | 2 | 7 | 258.25 | 0.13 | 6 |
| LjT | Secologanoside_qt | 79.21 | -1.53 | 0.09 | 1 | 6 | 226.20 | -1.89 | 4 |
| LjT | Secoxyloganin | 3.79 | -1.63 | 0.39 | 5 | 11 | 404.41 | -2.03 | 5 |
| LjT | Secoxyloganin_qt | 34.80 | -0.32 | 0.09 | 2 | 6 | 242.25 | -0.28 | 1 |
| LjT | Sitogluside | 20.63 | -0.14 | 0.62 | 4 | 6 | 576.95 | 6.34 | 9 |
| LjT | Stigmasterol | 43.83 | 1.44 | 0.76 | 1 | 1 | 412.77 | 7.64 | 5 |
| LjT | Stigmasterol-β-glucoside | 2.40 | -0.23 | 0.63 | 4 | 6 | 574.93 | 5.89 | 8 |
| LjT | Succinic acid | 29.62 | -0.44 | 0.01 | 2 | 4 | 118.10 | -0.41 | 3 |
| LjT | Sulcatone | 26.36 | 1.35 | 0.01 | 0 | 1 | 126.22 | 1.79 | 3 |
| LjT | Sweroside aglycone | 68.68 | 0.15 | 0.08 | 1 | 4 | 196.22 | 0.15 | 1 |
| LjT | Tetradecane | 15.94 | 1.79 | 0.04 | 0 | 0 | 198.44 | 6.76 | 11 |
| LjT | Thymol | 41.47 | 1.60 | 0.03 | 1 | 1 | 150.24 | 3.24 | 1 |
| LjT | Tricin | 27.86 | 0.51 | 0.34 | 3 | 7 | 330.31 | 2.30 | 3 |
| LjT | Ursolic acid | 16.77 | 0.67 | 0.75 | 2 | 3 | 456.78 | 6.47 | 1 |
| LjT | Vogeloside | 5.62 | -1.17 | 0.46 | 4 | 10 | 388.41 | -1.78 | 5 |
| LjT | WLN: NCR B1 | 55.48 | 1.61 | 0.02 | 0 | 1 | 117.16 | 2.19 | 0 |
| LjT | WLN: QR BQ DQ | 22.93 | 0.59 | 0.02 | 3 | 3 | 126.12 | 1.03 | 0 |
| LjT | WLN: VHR | 32.63 | 1.32 | 0.01 | 0 | 1 | 106.13 | 1.59 | 1 |
| LjT | XYLOSTOSIDINE | 43.17 | -1.07 | 0.64 | 4 | 9 | 415.51 | -1.66 | 4 |
| LjT | XYLOSTOSIDINE_qt | 4.01 | 0.34 | 0.15 | 1 | 4 | 253.35 | 0.09 | 1 |
| LjT | Zeaxanthin | 21.17 | 1.22 | 0.54 | 2 | 2 | 568.96 | 9.53 | 10 |
| LjT | ZINC03978781 | 43.83 | 1.32 | 0.76 | 1 | 1 | 412.77 | 7.64 | 5 |
| LjT | Zingiberene | 18.80 | 1.91 | 0.06 | 0 | 0 | 204.39 | 5.08 | 4 |
| LjT | α cadinene | 18.73 | 1.85 | 0.08 | 0 | 0 | 204.39 | 4.75 | 1 |
| LjT | α-Cubebene | 16.73 | 1.83 | 0.11 | 0 | 0 | 204.39 | 4.17 | 1 |
| LjT | β-Carotene | 37.18 | 2.25 | 0.58 | 0 | 0 | 536.96 | 12.00 | 1 |
| LjT | β-Citronellol | 38.89 | 1.20 | 0.02 | 1 | 1 | 156.30 | 3.05 | 5 |
| LjT | β-Cubebene | 32.81 | 1.83 | 0.11 | 0 | 0 | 204.39 | 4.22 | 1 |
| LjT | β-Rhodinol | 38.05 | 1.19 | 0.02 | 1 | 1 | 156.30 | 3.05 | 5 |
| LjT | γ-Muurolene | 21.53 | 1.84 | 0.08 | 0 | 0 | 204.39 | 4.80 | 1 |
| LjT | δ-Amorphene | 17.95 | 1.85 | 0.08 | 0 | 0 | 204.39 | 4.94 | 1 |
| LjT/AcT | Furol | 34.35 | 1.08 | 0.01 | 0 | 2 | 96.09 | 0.99 | 1 |
| LjT/AcT | Heriguard | 11.93 | -1.03 | 0.33 | 6 | 9 | 354.34 | -0.42 | 5 |
| LjT/AcT | Quercetin | 46.43 | 0.05 | 0.28 | 5 | 7 | 302.25 | 1.50 | 1 |
| LjT/AcT | Rutin | 3.20 | -1.93 | 0.68 | 10 | 16 | 610.57 | -1.45 | 6 |
| LjT/AcT | β-caryophyllene | 29.70 | 1.83 | 0.09 | 0 | 0 | 204.39 | 4.75 | 0 |
| LjT/AcT | β-Elemene | 25.63 | 1.84 | 0.06 | 0 | 0 | 204.39 | 4.79 | 3 |
| LjT/AcT/Cm | β-Sitosterol | 36.91 | 1.32 | 0.75 | 1 | 1 | 414.79 | 8.08 | 6 |
| LjT/Cm | Palmitic acid | 19.30 | 1.09 | 0.10 | 1 | 2 | 256.48 | 6.37 | 14 |
| AcT | (-)-nopinene | 44.84 | 1.80 | 0.05 | 0 | 0 | 136.26 | 2.93 | 0 |
| AcT | (2R,3R)-3,5-dihydroxy-2-(4-hydroxyphenyl)-7-methoxychroman-4-one | 24.84 | 0.14 | 0.26 | 3 | 6 | 302.30 | 2.00 | 2 |
| AcT | (E)-3-[4-hydroxy-3-[(E)-4-hydroxy-3-methyl-but-2-enyl]-5-(3-methylbut-2-enyl)phenyl]acrylic acid | 23.76 | 0.41 | 0.19 | 3 | 4 | 316.43 | 4.26 | 7 |
| AcT | (E)-3-[4-hydroxy-3-[(Z)-4-hydroxy-3-methyl-but-2-enyl]-5-(3-methylbut-2-enyl)phenyl]acrylic acid | 12.86 | 0.35 | 0.19 | 3 | 4 | 316.43 | 4.26 | 7 |
| AcT | (L)-alpha-Terpineol | 48.80 | 1.39 | 0.03 | 1 | 1 | 154.28 | 2.42 | 1 |
| AcT | 2-NONANONE | 8.51 | 1.34 | 0.02 | 0 | 1 | 142.27 | 2.70 | 6 |
| AcT | 4'-Methylcapillarisin | 72.18 | 0.57 | 0.35 | 2 | 7 | 330.31 | 3.06 | 6 |
| AcT | 5-Hydroxyferulate | 59.99 | 0.24 | 0.07 | 3 | 5 | 210.20 | 1.35 | 3 |
| AcT | 7-Methylcapillarisin | 5.08 | 0.51 | 0.34 | 2 | 7 | 330.31 | 3.06 | 4 |
| AcT | Arcapillin | 48.96 | 0.60 | 0.41 | 3 | 8 | 360.34 | 2.29 | 4 |
| AcT | Artepillin A | 68.32 | 0.45 | 0.24 | 2 | 4 | 316.43 | 3.82 | 6 |
| AcT | Artepillin C | 38.39 | 0.83 | 0.17 | 2 | 3 | 300.43 | 5.35 | 6 |
| AcT | Ayapanin | 41.55 | 0.97 | 0.06 | 0 | 3 | 176.18 | 1.88 | 1 |
| AcT | Azelex | 16.90 | -0.04 | 0.04 | 2 | 4 | 188.25 | 1.87 | 8 |
| AcT | Butal | 68.66 | 1.18 | 0.00 | 0 | 1 | 72.12 | 0.94 | 2 |
| AcT | Capillanol | 62.02 | 1.29 | 0.04 | 1 | 1 | 174.26 | 2.97 | 2 |
| AcT | Capillarin | 87.01 | 1.36 | 0.08 | 0 | 2 | 198.23 | 3.00 | 1 |
| AcT | Capillarisin | 57.56 | 0.49 | 0.31 | 3 | 7 | 316.28 | 2.81 | 3 |
| AcT | Capillarol | 37.41 | 0.54 | 0.12 | 1 | 4 | 260.31 | 3.17 | 5 |
| AcT | Capillene | 47.19 | 2.11 | 0.03 | 0 | 0 | 154.22 | 3.95 | 1 |
| AcT | Capillin | 52.44 | 1.50 | 0.04 | 0 | 1 | 168.20 | 3.38 | 1 |
| AcT | Car-3-ene | 45.15 | 1.85 | 0.04 | 0 | 0 | 136.26 | 2.87 | 0 |
| AcT | Cirsilineol | 4.81 | 0.77 | 0.37 | 2 | 7 | 344.34 | 2.55 | 4 |
| AcT | Cirsimaritin | 30.35 | 0.72 | 0.30 | 2 | 6 | 314.31 | 2.57 | 3 |
| AcT | Demethoxycapillarisin | 52.33 | 0.31 | 0.25 | 3 | 6 | 286.25 | 2.83 | 2 |
| AcT | D-limonene | 38.80 | 1.82 | 0.02 | 0 | 0 | 136.26 | 3.50 | 1 |
| AcT | Eugenol | 56.24 | 1.35 | 0.04 | 1 | 2 | 164.22 | 2.55 | 3 |
| AcT | Eupalitin | 46.11 | 0.62 | 0.33 | 3 | 7 | 330.31 | 2.01 | 3 |
| AcT | Eupatolitin | 42.55 | 0.16 | 0.37 | 4 | 8 | 346.31 | 1.74 | 3 |
| AcT | Genkwanin | 37.13 | 0.63 | 0.24 | 2 | 5 | 284.28 | 2.59 | 2 |
| AcT | Hirsutrin | 1.86 | -1.66 | 0.77 | 8 | 12 | 464.41 | -0.59 | 4 |
| AcT | Isoarcapillin | 57.40 | 0.40 | 0.41 | 3 | 8 | 360.34 | 2.29 | 4 |
| AcT | Isorhamnetin | 49.60 | 0.31 | 0.31 | 4 | 7 | 316.28 | 1.76 | 2 |
| AcT | Isorhamnetin-3-mono-β-D-glucoside | 4.11 | -1.35 | 0.80 | 7 | 12 | 478.44 | -0.34 | 5 |
| AcT | Isorhamnetin-3-O-glucoside | 1.17 | -1.24 | 0.80 | 7 | 12 | 478.44 | -0.34 | 5 |
| AcT | Isoscopoletin | 23.46 | 0.71 | 0.08 | 1 | 4 | 192.18 | 1.62 | 1 |
| AcT | Methyleugenol | 73.36 | 1.47 | 0.04 | 0 | 2 | 178.25 | 2.81 | 4 |
| AcT | Neocapillene | 24.45 | 2.09 | 0.03 | 0 | 0 | 154.22 | 3.95 | 0 |
| AcT | Norcapillene | 38.06 | 2.03 | 0.03 | 0 | 0 | 140.19 | 4.00 | 1 |
| AcT | OXL | 29.68 | -0.64 | 0.01 | 2 | 4 | 90.04 | -0.48 | 1 |
| AcT | Penta-1,3-diynylbenzene | 15.35 | 2.07 | 0.03 | 0 | 0 | 140.19 | 3.50 | 0 |
| AcT | Piceol | 36.80 | 0.87 | 0.03 | 1 | 2 | 136.16 | 1.30 | 1 |
| AcT | Rhamnocitrin | 12.90 | 0.48 | 0.27 | 3 | 6 | 300.28 | 2.02 | 2 |
| AcT | Salicylic acid | 32.13 | 0.63 | 0.03 | 2 | 3 | 138.13 | 1.17 | 1 |
| AcT | Scoparone | 74.75 | 0.85 | 0.09 | 0 | 4 | 206.21 | 1.87 | 2 |
| AcT | Scopoletin | 27.32 | 0.73 | 0.08 | 1 | 4 | 192.18 | 1.62 | 1 |
| AcT | Scopoletol | 27.77 | 0.71 | 0.08 | 1 | 4 | 192.18 | 1.62 | 1 |
| AcT | Vanillin | 52.00 | 0.68 | 0.03 | 1 | 3 | 152.16 | 1.31 | 2 |
| AcT | α-Humulene | 39.81 | 1.87 | 0.06 | 0 | 0 | 204.39 | 5.04 | 0 |
| Cm | (2R,3R,4S)-2-(6-aminopurin-9-yl)-4-(hydroxymethyl)oxolan-3-ol | 38.44 | -0.90 | 0.16 | 4 | 7 | 251.28 | -1.48 | 2 |
| Cm | (2R,3S,5S)-5-(6-aminopurin-9-yl)-2-(hydroxymethyl)oxolan-3-ol | 30.13 | -1.10 | 0.15 | 4 | 7 | 251.28 | -1.25 | 2 |
| Cm | 20-Hexadecanoylingenol | 28.20 | 0.30 | 0.68 | 3 | 6 | 586.94 | 7.38 | 17 |
| Cm | Adenine | 62.81 | -0.30 | 0.03 | 3 | 4 | 135.15 | -0.58 | 0 |
| Cm | Arachidonic acid | 45.57 | 1.20 | 0.20 | 1 | 2 | 304.52 | 6.41 | 14 |
| Cm | Caffeine | 89.46 | 0.58 | 0.08 | 0 | 5 | 194.22 | -0.10 | 0 |
| Cm | Cerevisterol | 39.52 | 0.35 | 0.77 | 3 | 3 | 432.76 | 5.26 | 4 |
| Cm | Cholesteryl palmitate | 31.05 | 1.45 | 0.45 | 0 | 2 | 625.19 | 14.35 | 21 |
| Cm | Cinnamaldehyde | 31.99 | 1.35 | 0.02 | 0 | 1 | 132.17 | 1.95 | 2 |
| Cm | CLR | 37.87 | 1.43 | 0.68 | 1 | 1 | 386.73 | 7.38 | 5 |
| Cm | Cordycedipeptide A | 63.12 | -0.34 | - | 4 | 6 | 227.30 | -0.65 | 4 |
| Cm | Cordycepin | 36.83 | -1.13 | - | 4 | 7 | 251.28 | -1.45 | 2 |
| Cm | Cordylagenin | 17.36 | 0.34 | 0.78 | 2 | 4 | 432.71 | 3.66 | 0 |
| Cm | D-Mannoheptulose | 29.74 | -2.02 | 0.05 | 6 | 7 | 210.21 | -3.20 | 6 |
| Cm | EIC | 41.90 | 1.16 | 0.14 | 1 | 2 | 280.50 | 6.39 | 14 |
| Cm | Ergosterol | 14.29 | 1.47 | 0.72 | 1 | 1 | 396.72 | 6.93 | 4 |
| Cm | Galactomannan | 10.92 | -3.92 | 0.70 | 11 | 16 | 504.50 | -6.01 | 7 |
| Cm | GLB | 47.71 | -1.89 | 0.04 | 5 | 6 | 180.18 | -2.51 | 1 |
| Cm | GUP | 43.04 | -1.82 | 0.04 | 5 | 6 | 180.18 | -2.51 | 1 |
| Cm | Isoergotamine | 8.10 | 0.32 | 0.21 | 3 | 9 | 581.73 | 3.12 | 4 |
| Cm | LFA | 8.46 | 1.83 | 0.13 | 0 | 0 | 282.62 | 9.50 | 17 |
| Cm | Linoleic | 41.90 | 1.23 | 0.14 | 1 | 2 | 280.50 | 6.39 | 14 |
| Cm | Linoleyl acetate | 42.10 | 1.36 | 0.20 | 0 | 2 | 308.56 | 6.85 | 16 |
| Cm | MTL | 17.73 | -1.58 | 0.03 | 6 | 6 | 182.20 | -2.94 | 5 |
| Cm | NCA | 71.13 | 0.44 | 0.02 | 2 | 3 | 122.14 | -0.32 | 1 |
| Cm | Nicotinic acid | 47.65 | 0.34 | 0.02 | 1 | 3 | 123.12 | 0.28 | 1 |
| Cm | Oleic acid | 33.13 | 1.14 | 0.14 | 1 | 2 | 282.52 | 6.84 | 15 |
| Cm | Peroxyergosterol | 44.39 | 0.86 | 0.82 | 1 | 3 | 428.72 | 6.73 | 4 |
| Cm | Stearic acid | 17.83 | 1.15 | 0.14 | 1 | 2 | 284.54 | 7.28 | 16 |
| Cm | Styrone | 38.35 | 1.14 | 0.02 | 1 | 1 | 134.19 | 1.69 | 2 |
| Cm | TGL | 15.13 | 0.54 | 0.13 | 0 | 6 | 891.67 | 22.26 | 56 |
| Cm | Thiamine | 19.87 | -0.32 | 0.11 | 3 | 4 | 265.40 | -0.05 | 4 |
| Cm | TRE | 2.32 | -3.08 | 0.24 | 8 | 11 | 342.34 | -4.26 | 4 |
| Cm | Uracil | 42.53 | 0.05 | 0.02 | 2 | 4 | 112.10 | -1.01 | 0 |
| Cm | Uralene | 11.70 | 0.63 | 0.49 | 4 | 7 | 384.41 | 3.43 | 4 |
| Cm | Uridine | 10.49 | -1.14 | 0.11 | 4 | 8 | 244.23 | -2.45 | 2 |
| Cm | Vitamin C | 13.34 | -0.86 | 0.04 | 4 | 6 | 176.14 | -1.76 | 2 |
| Cm | Vitamin G | 6.79 | -1.22 | 0.50 | 5 | 10 | 376.41 | 0.23 | 5 |

LjT, *Lonicera japonica* Thunberg; AcT, *Artemisia capillaris* Thunberg; Cm, *Cordyceps militaris*; MW, molecular weight;
OB, oral bioavailability; Caco-2, Caco-2 cell permeability; DL, drug-likeness score; Hdon, number of hydrogen bond donors;
Hacc, number of hydrogen bond acceptors; MW, molecular weight; AlogP, octanol-water partition coefficient log P;
RBN, number of rotatable bonds. **Supplementary Table S3. List of bioactive chemical components of FDY003.**

| Herbal medicines | Chemical components | OB | Caco-2 | DL | Hdon | Hacc | MW | AlogP | RBN |
| --- | --- | --- | --- | --- | --- | --- | --- | --- | --- |
| LjT | Chrysoeriol | 35.85 | 0.39 | 0.27 | 3 | 6 | 300.28 | 2.32 | 2 |
| LjT | Corymbosin | 51.96 | 0.88 | 0.41 | 1 | 7 | 358.37 | 2.80 | 5 |
| LjT | Eriodyctiol (flavanone) | 41.35 | 0.05 | 0.24 | 4 | 6 | 288.27 | 2.03 | 1 |
| LjT | Kaempferol | 41.88 | 0.26 | 0.24 | 4 | 6 | 286.25 | 1.77 | 1 |
| LjT | Luteolin | 36.16 | 0.19 | 0.25 | 4 | 6 | 286.25 | 2.07 | 1 |
| LjT/AcT | Quercetin | 46.43 | 0.05 | 0.28 | 5 | 7 | 302.25 | 1.50 | 1 |
| LjT/AcT/Cm | β-Sitosterol | 36.91 | 1.32 | 0.75 | 1 | 1 | 414.79 | 8.08 | 6 |
| AcT | 4'-Methylcapillarisin | 72.18 | 0.57 | 0.35 | 2 | 7 | 330.31 | 3.06 | 6 |
| AcT | Arcapillin | 48.96 | 0.60 | 0.41 | 3 | 8 | 360.34 | 2.29 | 4 |
| AcT | Artepillin A | 68.32 | 0.45 | 0.24 | 2 | 4 | 316.43 | 3.82 | 6 |
| AcT | Capillarisin | 57.56 | 0.49 | 0.31 | 3 | 7 | 316.28 | 2.81 | 3 |
| AcT | Cirsimaritin | 30.35 | 0.72 | 0.30 | 2 | 6 | 314.31 | 2.57 | 3 |
| AcT | Eupalitin | 46.11 | 0.62 | 0.33 | 3 | 7 | 330.31 | 2.01 | 3 |
| AcT | Eupatolitin | 42.55 | 0.16 | 0.37 | 4 | 8 | 346.31 | 1.74 | 3 |
| AcT | Genkwanin | 37.13 | 0.63 | 0.24 | 2 | 5 | 284.28 | 2.59 | 2 |
| AcT | Isoarcapillin | 57.40 | 0.40 | 0.41 | 3 | 8 | 360.34 | 2.29 | 4 |
| AcT | Isorhamnetin | 49.60 | 0.31 | 0.31 | 4 | 7 | 316.28 | 1.76 | 2 |
| Cm | Cordycepin | 36.83 | -1.13 | - | 4 | 7 | 251.28 | -1.45 | 2 |

LjT, *Lonicera japonica* Thunberg; AcT, *Artemisia capillaris* Thunberg; Cm, *Cordyceps militaris*; MW, molecular weight;
OB, oral bioavailability; Caco-2, Caco-2 cell permeability; DL, drug-likeness score; Hdon, number of hydrogen bond donors;
Hacc, number of hydrogen bond acceptors; MW, molecular weight; AlogP, octanol-water partition coefficient log P;
RBN, number of rotatable bonds.

**Supplementary Table S4. List of targets of active phytochemical ingredients of FDY003.**

| Herbal medicines | Chemical components | Targets |
| --- | --- | --- |
| LjT | Chrysoeriol | ABCC1*, CREB1, CYP1B1*, PTPRS, XDH |
| LjT | Corymbosin | ABCG2*, CREB1, CYP1B1*, PTPRS |
| LjT | Eriodyctiol (flavanone) | ABCB1, ABCC1*, ADIPOQ, APOB, BDNF, CA12, CA4, CA7, CBR1*, CCK, CCL2, CD69, CD86, CYP19A1*, CYP1A2*, CYP1B1*, CYP3A4*, HMGA1, HMGCS2, HMOX1, HSD17B1, KCNH2, KCNMA1, LDLR, PGF, PPARA, RAPGEF1, SCD, SCD5, SHBG*, TAS2R31, TLR2 |
| LjT | Kaempferol | ABCB1*, ABCC1*, ABCG2*, AHR*, AKR1B1, AKT1, ALOX12, ALOX5, AR*, ATM, BCHE, CA12, CA2*, CA7, CASP3, CASP9, CCL2, CDK1*, CDK2, CHUK, CISD1, CSF2, CTDSP1, CYP1A1, CYP1A2*, CYP1B1*, CYP2B6, CYP2D6*, CYP3A4, DAPK1*, DIO2, EGFR*, ESR1*, ESR2, ESRRA, F2*, FLT3, GSTP1, H2AFX, HCK, HMOX1, HSD17B1, HSD17B2, IGF1R, IGF2, IL2, ITGA2, JUN, MAPK1, MAPK3, MMP1, MMP2, MPO*, NFKBIA, NOS1, NOS2, NOX4, NR1I2*, NR1I3, P4HB, PIM1, PTGES, PTPRS, RB1, RPS6KA3, SLC2A1*, SRC, STAT1, STAT3, TNFRSF11B, TP53, TYR*, UGT1A1, UGT1A10, UGT1A3, UGT1A7, UGT1A8, UGT1A9, UGT3A1, XDH |
| LjT | Luteolin | ABCC1, ABCG2*, ADAMTS3, ADAMTS4, ADORA1, AGT, AKR1B1, AKR1B10*, AKT1, ALOX15, ALOX5, APP, ARG1*, AURKB, BCL2L1, C3, CA12, CA2*, CA4, CA7, CASP3*, CASP7, CASP9, CCNA2, CCNB1*, CCNB2*, CCNB3, CD38, CDH1, CDK1*, CDK2*, CDK4, CDK5R1, CDK6*, CREB1, CSNK2A1, CSNK2A2, CSNK2B, CYP19A1, CYP1A1, CYP1A2*, CYP1B1*, E2F5, EGFR, ERBB2, ESR2, EZH2, FLT3, FN1, FOS*, FOSB, FOXO1, GLO1, GPR35, GSK3B*, HMOX1, HSP90AA1, IGF1, IGF2, IL1B, IRS1, JUN*, JUNB, JUND, LCN2, MAOA, MAP3K8, MAPK1, MAPK10, MAPK3, MAPK8, MAPK9, MMP12, MMP2*, MMP9*, MTOR, NFE2L2, NOS1, NOS2, NOX4, PARP1*, PCK1, PKM, PPARG, PTK2, PTPRS, RPS6KA1, RPS6KA2, RPS6KA3, SMAD2, STAT3, SYK, TAF9, TBK1, TLR4, TLR5, TNFRSF10B, TNKS, TNKS2, TOP1*, TP53, TTR, UGT1A3, USP8, VEGFA, VRK1, XDH |
| LjT/AcT | Quercetin | ABCA1, ABCB1*, ABCC1*, ABCC4, ABCC5, ABCG2*, ACACA, ADIPOQ, ADORA1, ADORA2A, AHR, AIFM1, AKR1A1*, AKR1B1, AKR1C1, AKR1C2*, AKR1C3, AKR1C4, AKT1*, ALK*, ALOX12, ALOX15, ALOX5, AOX1, APAF1, APOB, AR*, ARNT, ATP2A1, ATP5A1, ATP5B, ATP5C1, AURKB*, AVPR2, AXL*, BACE1, BAX, BCL2, BDNF, BID, CA1, CA12, CA13, CA14, CA2*, CA3, CA4, CA5A, CA6, CA7, CA9*, CAMK2B, CASP3*, CASP7, CASP8, CASP9*, CAT, CCL2*, CCR4*, CD38, CD97, CDK1*, CDK2, CDKN1A, CDKN2A, CFTR, CHEK2, CHUK, CKB, CSF2*, CSNK2A1, CTNNB1, CTRL, CXCL10, CXCR1*, CXCR4, CYBB, CYCS, CYP19A1*, CYP1A1*, CYP1A2*, CYP1B1*, CYP2C8*, CYP2C9, CYP2D6, CYP2E1, CYP3A4*, DAPK1*, DIABLO, DIO2, DRD4, EDN1, EEF1A1, EGFR*, EIF2A, EIF2AK2, ELAVL1*, ERN1, ERN2, ESR1, ESR2, F2*, F3, FAU, FLT3, FNDC5, FOS, FOXM1, GADD45A, GLO1, GLRA1, GPR35, GSK3B*, GSTP1*, HCK, HIBCH, HIF1A*, HIST3H3, HMOX1, HPGDS, HSD17B2, HSPA1A, HSPA4, HSPB1, ICAM1, IGF1R*, IGFBP3, IL15, IL17A, IL1B, IL6*, IL8, JUN*, KCNMA1, KDR*, KRAS, LEPR, MAOA, MAPK1, MAPK14, MAPK3, MAPK8*, MCL1*, MET*, MMP1, MMP13*, MMP2*, MMP3*, MMP9*, MPO*, NAMPT, NEK2*, NEK6, NFE2L2, NFKBIA, NKX3-1, NOS1, NOS2, NOS3, NOX3, NOX4, NPC1L1, NPY1R, NR1I2, NR1I3, NT5E, NUAK1*, ODC1, P4HB, PARP1, PDX1, PIK3CA, PIK3CG*, PIK3R1*, PIM1, PKN1, PLA2G1B, PLAT, PLAU, PLK1*, PON1, PON2, POR, PPARA, PRDX5, PTGS1, PTGS2*, PTK2*, PTPRS, PYGB, PYGL*, PYGM, RAF1, RB1, RNASEL, RPS6KA5, SERPIND1, SESN2, SIRT1*, SLC12A2, SLC2A1, SLC2A2*, SLC2A4, SP1, SRC*, STAT1, STK17B, SULT1A1, SULT1E1, TBK1, TFAM, TGFA, TGM2, TLR1, TLR10, TLR2, TLR4, TLR6, TMPRSS11D, TNF, TNFRSF10B, TNFSF10, TP53*, TRPM7, TYR, UGT1A1, UGT1A10, UGT1A3, UGT1A5, UGT1A7*, UGT1A8, UGT1A9, UGT2A3, UGT2B15, UGT2B4, UGT3A1, VEGFA*, XBP1, XDH |
| LjT/AcT/Cm | β-Sitosterol | ACBD7, CASP3, CASP9, CYP17A1*, DHPS, DRAP1, GPBAR1, ICAM1, LFT, MDM2*, MEX3D, MTOR*, NPC1L1, NR1D2, PARP1, RAC1*, RANBP2, RUVBL1, TNNC1, USF1* |
| AcT | 4'-Methylcapillarisin | CREB1 |
| AcT | Arcapillin | CREB1, CYP1B1*, PTPRS |
| AcT | Artepillin A | CPSF4, CYP2R1*, EME1, FOLH1, PTPRJ*, RNF31 |
| AcT | Capillarisin | CREB1, CYP1B1*, PTPRS |
| AcT | Cirsimaritin | ADORA1, ADORA2A, ADORA3*, AKR1B1, CREB1, CYP1B1*, PTPRS |
| AcT | Eupalitin | CREB1, CYP1B1* |
| AcT | Eupatolitin | CREB1, CYP1B1* |
| AcT | Genkwanin | ALDH2*, CREB1, CYP1A1, CYP1A2, CYP1B1*, DUSP1, PTPRS, XDH |
| AcT | Isoarcapillin | CREB1, PTPRS |
| AcT | Isorhamnetin | ABCB1, ABCC1, AKT1, CA12, CA2*, CA4, CA7, CREB1, CYP1A1*, CYP1B1*, ERN1*, HMOX1, MAPK8, MAPK9, NOS2, P4HB, PON2, PRKCD, XDH |
| Cm | Cordycepin | ADCY5, ADK, ADORA1, ADORA2A, ADORA2B, ADORA3*, AHCY*, AHCYL1, AHCYL2, AMD1, BCL7A, CASP3, CASP8, CASP9*, DCK, DGUOK, DNMT3B*, DOT1L, DTYMK, FOXP3, GAPDH*, HGF, HSPA5*, HSPA8, IL10, IL1B, IL6, IMPDH1, KMT5C, LEPR, MMP9, MTAP, MYC, NOS1, P2RX1, P2RY11, PAPOLA, PRMT7, QARS1, RARS1, RNASEL*, SETDB1, SLC29A1, SMS, SRM, STAR, TARS1, TK1*, TLR4, TMPRSS11D |

LjT, *Lonicera japonica* Thunberg; AcT, *Artemisia capillaris* Thunberg; Cm, *Cordyceps militaris*.

*, liver cancer-related targets.

**Supplementary Table S5. List of the genes and proteins associated with the pathological mechanisms of liver cancer.**

| A1ATD, A2M, AADAT, ABCB1*, ABCB11, ABCB1B, ABCB4, ABCB6, ABCC1*, ABCC2, ABCC3, ABCG2*, ABL1, ABO, ACACA, ACE, ACLY, ACNINV1, ACO2, ACOT1, ACOX1, ACSL4, ACTB, ACTBL2, ACTC1, ACTG1, ADAM17, ADAMTS1, ADAMTS13, ADAMTS17, ADD1, ADGRG7, ADH1B, ADH4, ADIPOQ, ADORA3*, ADRA1A, ADRA2B, AFAP1-AS1, AFP, AHCY*, AHR*, AIP, AKR1A1*, AKR1B10*, AKR1C2*, AKT1*, AKT2, AKT3, ALAS2, ALB, ALDH1L1, ALDH2*, ALGS1, ALK*, ALPS, AMACR, ANGPT1, ANGPT2, ANGPTL6, ANGPTL8, ANLN, ANXA2, ANXA4, ANXA5, ANXA7, APC, APCS, APEX1, APLN, APOA1, APOA4, APOBEC1, APOBEC3A, APOBEC3B, APOC2, APOF, APRT, AQP9, AR*, AREG, ARG1*, ARHGAP11A, ARHGEF39, ARID1A, ARID1B, ARID2, ARL5A, ARMC10, ASAP3, ASF1B, ASH1L, ASNS, ASPG, ASPH, ASPM, ASS1, AT, ATF3, ATF6, ATG5, ATG7, ATM, ATP11C, ATP5ME, ATP5PD, ATP7A, ATP7B, ATP8B1, AURKA, AURKB*, AXIN1, AXIN2, AXL*, AXUD1, B2M, BAD, BAK1, BANCR, BAP1, BARD1, BATF2, BAX, BBC3, BCAS3, BCL10, BCL2, BCL2L1, BCL2L11, BCO2, BDCHS, BECN1, BID, BIRC2, BIRC3, BIRC5, BMI1, BMP2, BMP6, BMPER, BMPR1A, BRAF, BRCA1, BRCA2, BRD4, BRIP1, BSG, BTBD11, BTBD7, BTG2, BUB1, BUB1B, C11orf65, C14orf180, C1QTNF6, C2, C9, CA2*, CA9*, CADM1, CALB2, CALCA, CAP2, CAR3, CASC15, CASC2, CASP10, CASP3*, CASP7, CASP8, CASP9*, CAT, CAV1, CBR1*, CBS, CCAT1, CCAT2, CCBE1, CCDC134, CCEPR, CCL14, CCL2*, CCL20, CCL3, CCL5, CCN1, CCN2, CCNA1, CCNA2, CCNB1*, CCNB2*, CCND1, CCND2, CCNDBP1, CCNE1, CCNF, CCNG1, CCNH, CCR1, CCR4*, CCR5, CCR6, CD163, CD24, CD274, CD276, CD34, CD40, CD44, CD5L, CD63, CD80, CD81, CD82, CDC20, CDC25A, CDC25C, CDC45, CDC6, CDC73, CDCA2, CDCA3, CDCA5, CDCA8, CDH1, CDH13, CDH17, CDH2, CDH3, CDH6, CDK1*, CDK14, CDK2*, CDK4, CDK5RAP3, CDK6*, CDKN1A, CDKN1B, CDKN1C, CDKN2A, CDKN2B, CDKN2B-AS1, CDKN2C, CDKN3, CDT1, CDX2, CEACAM3, CEACAM5, CEBPA, CEBPB, CEBPD, CELSR3, CENPA, CENPE, CENPF, CENPM, CENPU, CENPW, CEP131, CEP55, CERS2, CETP, CFAP52, CFLAR, CFP, CFTR, CGREF1, CHAF1B, CHD1L, CHEK1, CHEK2, CHGA, CHN2, CHP2, CIB1, CIDEA, CKAP2L, CLDN10, CLDN7, CLEC1B, CLEC4G, CLEC4M, CLSTN2, CLTC, CLTRN, CLU, CNDP1, CNDP2, CNOT9, CNTNAP4, COL15A1, COL18A1, COLEC10, COMT, COPS5, COX7B2, COX8A, CP, CPEB3, CPN2, CPQ, CPT1B, CRABP1, CRBN, CREBBP, CRHBP, CRHR2, CRNDE, CRP, CRY1, CRYL1, CSF2*, CSF3, CSK, CSMD1, CSNK1A1, CSPG4, CSRNP1, CTAG1B, CTLA4, CTLN2, CTNNA1, CTNNB1, CTNND1, CTSD, CTTN, CXCL1, CXCL12, CXCL14, CXCL16, CXCL8, CXCL9, CXCR1*, CXCR4, CXCR6, CYCS, CYGB, CYLD, CYP17A1*, CYP19A1*, CYP1A1*, CYP1A2*, CYP1B1*, CYP2A4, CYP2A6, CYP2B6, CYP2C19, CYP2C29, CYP2C8*, CYP2D6*, CYP2E1, CYP2F2, CYP2R1*, CYP39A1, CYP3A16, CYP3A23-3A1, CYP3A4*, CYP3A5, CYP4A11, CYP7A1, CYTOR, DACT1, DACT2, DANCR, DAPK1*, DBH, DCAF4L2, DCC, DCD, DCN, DDB2, DDR1, DDR2, DDX54, DEPDC1, DEPDC1B, DEPDC5, DERL2, DGCR5, DHCR7, DIABLO, DICER1, DIPK2B, DIRAS3, DKK1, DKK4, DLC1, DLEC1, DLGAP5, DNAJA3, DNAJB1, DNAJC6, DNASE1L3, DNMT1, DNMT3A, DNMT3B*, DPP10, DPYD, DTL, DVL1, DVL2, DYNLRB1, DYNLRB2, E2F1, E2F2, E2F3, E2F8, EAF2, ECI2, ECM1, ECT2, EDC4, EFNB2, EGF, EGFR*, EGFR-AS1, EGLN3, EGR1, EGR2, EHD3, EIF2D, EIF3H, EIF4E, EIF4EBP1, ELAVL1*, ELK1, ENG, ENO1, ENO2, ENSG00000216663, EP300, EPAS1, EPCAM, EPHA2, EPHB2, EPHX1, EPO, EPS8L3, ERBB2, ERBB3, ERBB4, ERCC1, ERCC2, ERCC3, ERCC4, ERCC5, ERCC6, ERGIC3, ERN1*, ERP29, ESM1, ESR1*, ESR2, ETFA, ETS1, ETV6, EXO1, EXOSC2, EZH2, EZR, F11, F12, F2*, FABP1, FABP5, FAH, FAM111B, FAM134B, FAM180A, FAM65C, FAM72B, FAM83D, FANCC, FANCD2, FANCI, FAS, FASLG, FASN, FATE1, FBN1, FBP1, FBXL18, FBXW7, FCN2, FCN3, FDFT1, FEN1, FGF19, FGF2, FGF3, FGF4, FGFR1, FGFR2, FGFR3, FGFR4, FGL1, FHIT, FKSG49, FLCN, FLT1, FLT4, FLVCR1, FN1, FOS*, FOSB, FOXA1, FOXA2, FOXE1, FOXM1, FOXO1, FOXO3, FOXP3, FRZB, FST, FTX, FZD7, G6PC1, GAB1, GABRD, GADD45B, GADD45G, GADL1, GALNT14, GAPDH*, GART, GAS5, GAST, GBA3, GCKR, GDF15, GDF2, GGT1, GHET1, GHR, GHRL, GINS1, GJB1, GJB2, GLB1, GLDN, GLI1, GLUL, GLYAT, GMNN, GNAO1, GNAS, GNAZ, GNMT, GOLM1, GPC3, GPD1, GPER1, GPM6A, GPR158, GPR182, GPT, GPX1, GPX2, GPX3, GRAMD1A, GRB2, GREM1, GRIK2, GRP, GSD1A, GSD1B, GSD1C, GSD9C, GSK3B*, GSTA1, GSTM1, GSTM3, GSTO1, GSTO2, GSTP1*, GSTT1, GTSE1, GYS2, H19, H2AC18, HABP2, HAGLR, HAMP, HAO2, HAVCR2, HBA2, HCC, HCCAT5, HCCS, HCFC1, HCP5, HDAC1, HDAC10, HDAC4, HDGF, HEBP2, HEIH, HEPACAM, HEPN1, HERC2, HERC3, HERC5, HFE, HFE1, HGF, HGFAC, HHCM, HHIP, HIF1A*, HIGD2A, HIGM1, HJURP, HK2, HLA-A, HLA-B, HLA-C, HLA-DPA1, HLA-DPB1, HLA-DQA1, HLA-DQB1, HLA-DQB2, HLA-DRA, HLA-DRB1, HLA-G, HLF, HMGA2, HMGB1, HMGB2, HMGCR, HMMR, HMOX1, HNF1A, HNF1A-AS1, HNF1B, HNF4A, HOTAIR, HOTTIP, HOXA10, HOXA11-AS, HOXA13, HOXB13, HOXD9, HPAFP, HPSE, HRAS, HSD3B2, HSDL2, HSP90AA1, HSPA1B, HSPA4, HSPA5*, HSPA9, HSPB1, HTATIP2, HTGTI, HULC, IAP, ICAM1, IDH1, IDH2, IFI27, IFNA1, IFNA2, IFNAR1, IFNAR2, IFNB1, IFNG, IFNL3, IFNL4, IFT88, IGBP1, IGDCC4, IGF1, IGF1R*, IGF2, IGF2BP1, IGF2BP2, IGF2BP3, IGF2R, IGFALS, IGFBP3, IKBKG, IL10, IL12A, IL12B, IL12RB1, IL17A, IL17D, IL18, IL1A, IL1B, IL1RAP, IL1RN, IL2, IL21, IL23R, IL27, IL28B, IL4, IL6*, IL6R, IL7, IL8, ING1, ING2, ING3, INMT, INPP4B, INS, INSIG1, INSIG2, INS-IGF2, INSR, IQGAP1, IQGAP2, IQGAP3, IRF1, IRF2, IRGM, IRS1, IRS2, ISG15, ITGA6, ITGAV, ITGB1, ITGB4, ITIH1, ITIH3, JAG1, JAK1, JAK2, JDP2, JUN*, JUP, KAT2B, KBTBD11, KCNN2, KCNQ1OT1, KDM4C, KDM8, KDR*, KEAP1, KIF11, KIF14, KIF15, KIF18A, KIF18B, KIF1B, KIF20A, KIF23, KIF2C, KIF4A, KIFC1, KIR2DL1, KIR2DL2, KIR2DL3, KIR2DL4, KIR2DS2, KIR3DL1, KIR3DL2, KIR3DS1, KIT, KITLG, KLF4, KLF6, KLK3, KLRK1, KMT2A, KMT2B, KMT2C, KMT5A, KRAS, KRT14, KRT18, KRT19, KRT20, KRT5, KRT7, KRT8, LAMC2, LAMTOR5, LAPTM4B, LARGE1, LARP1, LCAT, LCO, LDHA, LEF1, LEPR, LETM1, LGALS3, LHPP, LIFR, LILRB5, LIN28B, LINC00221, LINC00261, LINC01018, LINC-ROR, LINGO2, LMNA, LNCRNA-ATB, LOC110806263, LOC727677, LOX, LPA, LRAT, LRP6, LRRC1, LRRC56, LRRC59, LTA, LTF, LYVE1, LZTS1, MACIR, MACROD2, MAD1L1, MAD2L1, MAGEA1, MAGEA3, MAGEA4, MAGEA6, MAGEC2, MALAT1, MAP2K1, MAP3K1, MAP3K8, MAPK1, MAPK14, MAPK3, MAPK8*, MAPT, MARCHF8, MARCO, MARK4, MASP2, MAT1A, MAX, MBL2, MBOAT7, MBTPS1, MBTPS2, MCC, MCL1*, MCM10, MCM2, MCM7, MCM8, MDK, MDM2*, ME1, MECOM, MECP2, MED1, MEG3, MELK, MEN1, MEOX2, MET*, MFGE8, MFSD2A, MGAT5, MGMT, MICA, MIF, MIR100, MIR101-1, MIR106A, MIR106B, MIR107, MIR10A, MIR10B, MIR122, MIR122A, MIR124-1, MIR125A, MIR125B1, MIR126, MIR127, MIR128-1, MIR128-2, MIR129-1, MIR130A, MIR130B, MIR132, MIR133A1, MIR133B, MIR137, MIR138-1, MIR139, MIR140, MIR141, MIR142, MIR143, MIR144, MIR145, MIR146A, MIR146B, MIR148A, MIR148B, MIR149, MIR150, MIR151, MIR152, MIR155, MIR15A, MIR15B, MIR16-1, MIR16-2, MIR17, MIR181A1, MIR181A2, MIR181B1, MIR181B2, MIR181C, MIR181D, MIR182, MIR183, MIR185, MIR186, MIR18A, MIR191, MIR192, MIR193A, MIR193B, MIR195, MIR196A1, MIR196A2, MIR196B, MIR197, MIR199A1, MIR199B, MIR19A, MIR200A, MIR200B, MIR200C, MIR203A, MIR204, MIR205, MIR206, MIR207, MIR20A, MIR21, MIR210, MIR212, MIR214, MIR215, MIR218-1, MIR219A1, MIR22, MIR221, MIR222, MIR223, MIR224, MIR23A, MIR23B, MIR24-1, MIR24-2, MIR25, MIR26A, MIR26A1, MIR26B, MIR27A, MIR290, MIR292, MIR296, MIR29A, MIR29C, MIR300, MIR301A, MIR30A, MIR30B, MIR30D, MIR30E, MIR31, MIR320A, MIR324, MIR331, MIR335, MIR336, MIR338, MIR339, MIR34, MIR340, MIR342, MIR347, MIR349, MIR34A, MIR34B, MIR34C, MIR350, MIR352, MIR370, MIR372, MIR373, MIR375, MIR383, MIR409, MIR424, MIR429, MIR449A, MIR451A, MIR483, MIR486-1, MIR490, MIR499, MIR499A, MIR502, MIR503, MIR505, MIR519D, MIR520B, MIR520C, MIR520G, MIR539, MIR584, MIR598, MIR608, MIR615, MIR652, MIR671, MIR708, MIR760, MIR872, MIR885, MIR9-1, MIR92B, MIR93, MIR9-3, MIR96, MIR98, MIR99A, MIR99B, MIRLET7A1, MIRLET7A3, MIRLET7B, MIRLET7C, MIRLET7D, MIRLET7E, MIRLET7G, MIRLET7I, MITF, MKI67, MKNK1, MKNK2, MLH1, MLH3, MLXIPL, MMD, MME, MMP1, MMP11, MMP13*, MMP14, MMP2*, MMP3*, MMP7, MMP9*, MOGAT2, MPEG1, MPO*, MRE11, MRO, MRPL3, MSH2, MSH3, MSH5, MSH6, MSLN, MSMB, MSR1, MSRA, MST1R, MT1A, MT1E, MT1F, MT2A, MTA1, MTBP, MT-CO1, MT-CYB, MTDH, MTHFR, MTOR*, MTR, MTRR, MTUS1, MUC1, MUC16, MUC2, MUC4, MUC5AC, MUTYH, MVK, MXI1, MYB, MYBL2, MYC, MYCBP, MYCN, MYO5B, NAA40, NAFLD1, NANOG, NAT1, NAT2, NBN, NBPF12, NCAM1, NCAPG, NCAPH, NCOA4, NCOA5, NCOR1, NDC80, NDRG1, NDUFA13, NEAT1, NEIL3, NEK2*, NF1, NFE2L2, NFKB1, NFKBIA, NFRSF10B, NKILA, NKX2-1, NKX2-8, NLRC3, NME1, NNMT, NONO, NOP16, NORAD, NOS2, NOS3, NOTCH1, NOTCH2, NOTCH3, NPTN-IT1, NQO1, NQO2, NR0B2, NR1H2*, NR1H4, NR1I2, NR1I3, NRAS, NTRK1, NTRK3, NUAK1*, NUDCD3, NUDT7, NUF2, NUS1, NUSAP1, NUTM1, OAT, ODZ1, OGG1, OIT3, OLFML2A, OLFML2B, ORC1, OSBPL1A, OSR1, PALB2, PAMR1, PANDAR, PARK2, PARK7, PARP1*, PAX8, PBK, PBRM1, PCAT1, PCK1, PCLAF, PCLO, PCNA, PDCD1, PDGFB, PDGFRA, PDGFRB, PDGFRL, PDIA3, PDK4, PDPN, PDRG1, PEG10, PER1, PER3, PFIC1, PFIC2, PFIC4, PGD, PGK1, PGR, PHB, PHF19, PHF20, PHGDH, PHLDA1, PIGR, PIK3CA, PIK3CB, PIK3CD, PIK3CG*, PIK3R1*, PIK3R2, PIK3R3, PIN1, PINX1, PIP, PITPNM3, PITX1, PK, PKHD1, PKM, PKMYT1, PKP1, PKP3, PLA2G2A, PLAC8, PLAG1, PLAU, PLAUR, PLCE1, PLCG1, PLK1*, PLPP5, PLS1, PLVAP, PLXDC1, PML, PMS1, PMS2, PNPLA3, PNPO, PNPT1, PODXL, POLD1, POLE, POLK, POLR2E, POU5F1, PPARA, PPARG, PPAT, PPM1D, PPP1R1A, PPP2R1B, PPP4R3C, PQLC3, PRC1, PRDM2, PRDM5, PRDX1, PRDX2, PRDX6, PRECSIT, PRICKLE2, PRKACA, PRKAR1A, PRKCA, PRKCB, PRKCD, PRKCE, PRKDC, PRKN, PROM1, PSD3, PSMA4, PSMB8, PSMD10, PSMG2, PTCH1, PTCSC1, PTEN, PTENP1, PTER, PTGS2*, PTH1R, PTHLH, PTK2*, PTK2B, PTPN11, PTPN12, PTPN3, PTPRC, PTPRH, PTPRJ*, PTPRO, PTTG1, PVT1, PYCARD, PYGL*, PZP, RAB10, RAB1B, RAB22A, RAB24, RAB25, RAB4B, RABL3, RAC1*, RACGAP1, RAD50, RAD51, RAD51C, RAD51D, RAD51L3-RFFL, RAD54B, RAD54L, RAF1, RAN, RARA, RARB, RARG, RASA1, RASGEF1A, RASSF1, RB1, RB1CC1, RBM39, RBMY, RBP5, RCAN1, RCHY1, RECK, REG3A, RELA, RET, RGN, RHOA, RHOC, RIOX2, RJALS, RNASEL*, RND3, RNF157, RNF180, RNF6, RNMTL1, ROBO1, RORA, RORC, ROS1, RPL36A, RPL9, RPS23, RPS28, RPS6, RPS6KA3, RPS6KB1, RRAS2, RRM1, RRM2, RSF1, RSPO1, RSPO3, RTP3, RUNX3, RXRA, S100A1, S100A4, SALL4, SAR1A, SAR1B, SARNP, SART1, SASH1, SCAP, SCARA5, SCD, SCT, SDF2L1, SDHA, SDHB, SDHC, SDHD, SERPINA1, SERPINA3, SERPINA4, SERPINB3, SERPINB5, SERPINE1, SET, SETD2, SF3B1, SF3B2, SFN, SFPQ, SFRP1, SHBG*, SHC1, SHH, SHISA5, SHMT1, SIRT1*, SIX3, SKA1, SKA3, SKP2, SLC10A1, SLC10A2, SLC11A2, SLC15A2, SLC17A5, SLC22A1, SLC22A10, SLC22A18, SLC25A13, SLC25A47, SLC26A6, SLC2A1*, SLC2A2*, SLC31A1, SLC37A4, SLC38A9, SLC40A1, SLC4A2, SLC51A, SLC5A5, SLCO1B1, SLCO1B3, SLIT2, SMAD2, SMAD3, SMAD4, SMAD7, SMARCA2, SMARCA4, SMARCB1, SMARCE1, SMO, SMYD3, SNAI1, SNAI2, SNHG1, SNHG11, SNHG12, SNHG15, SNHG20, SNX10, SOCS1, SOCS2, SOCS3, SOD1, SOD2, SOS1, SOX2, SOX2-OT, SOX4, SOX9, SP1, SPARC, SPATA21, SPC25, SPHK2, SPP1, SPRTN, SPRY4-IT1, SQLE, SQSTM1, SRC*, SRD5A2, SREBF1, SREBF2, SRGAP1, SRMS, SRPX, SRRD, SRS1, SSRP1, SST, SSX1, ST14, ST3GAL6, STAB2, STAC3, STARD13, STARD8, STAT1, STAT3, STAT4, STEAP3, STK11, STMN1, SUFU, SULF1, SULT1A1, SYP, TACC3, TAGLN2, TALDO1, TATDN1, TBX3, TCF19, TCF4, TCF7, TCF7L2, TCIM, TCP10L, TEDC2, TEK, TEKT5, TEP1, TERC, TERF1, TERT, TFCP2, TFDP3, TFE3, TFEB, TFF1, TFPI2, TFRC, TG, TGFA, TGFB1, TGFB2, TGFB3, TGFBR1, TGFBR2, TGFBR3, TGM3, TH, THBS1, THEM4, THY1, TICRR, TIMD4, TIMP1, TIMP2, TIMP3, TJP2, TK1*, TLR2, TLR3, TLR4, TLR7, TLR9, TM4SF5, TM6SF2, TMEM132D, TMEM176A, TMEM45A, TMEM70, TNF, TNFRSF10A, TNFRSF10B, TNFRSF12A, TNFRSF6B, TNFRSF9, TNFSF10, TNFSF9, TNK2, TONSL, TOP1*, TOP2A, TOP2B, TOX3, TP53*, TP63, TP73, TPX2, TRA, TRAIP, TRB, TRD, TRG, TRIM24, TRIM35, TRIM52AS1, TRIO, TRIP13, TRMT11, TROAP, TRP53, TSC1, TSC2, TSC22D1, TSLP, TST, TTC36, TTC39A, TTK, TTLL9, TUG1, TUSC7, TWIST1, TXNDC5, TXNRD1, TYMP, TYMS, TYR*, TYRSN1, UBD, UBE2C, UBE2E2, UBE2T, UCA1, UCHL1, UGDH, UGT1A1, UGT1A10, UGT1A6, UGT1A7*, UGT1A9, UHRF1, UMPS, URGCP, URI1, UROC1, USF1*, USP2, VANGL1, VASN, VCAM1, VDR, VEGFA*, VEGFC, VEGFD, VHL, VIM, VIP, VIPR1, VKORC1, VPS37A, VSIG4, VWCE, WDR17, WDR62, WDR76, WFDC1, WND, WNT1, WNT2, WNT3, WNT3A, WNT5A, WRAP53, WRN, WSCD1, WSPAR, WT1, WWOX, WWTR1, XAGE1B, XIAP, XIST, XPA, XPC, XPO5, XRCC1, XRCC2, XRCC3, XRCC4, XRCC5, YAP1, YIF1A, YTHDF2, YY1AP1, ZC4H2, ZDHHC2, ZEB1, ZEB1AS1, ZEB1-AS1, ZEB2, ZFAS1, ZFHX3, ZFP36, ZG16, ZIC2, ZIC4, ZIC5, ZNF23, ZNF689, ZNF71, ZNRD1, ZNRD1-AS1, ZNRD1ASP, ZSCAN22, ZWINT |
| --- |

*, Target genes and proteins of FDY003.

**Supplementary Table S6. Binding energies between the bioactive chemical components of FDY003 and their targets.**

| Chemical components | Targets | Binding energies |
| --- | --- | --- |
| Kaempferol | AR | −8.0 kcal/mol |
| Kaempferol | EGFR | −8.1 kcal/mol |
| Kaempferol | ESR1 | −8.6 kcal/mol |
| Luteolin | JUN | −8.8 kcal/mol |
| Quercetin | AKT1 | −6.6 kcal/mol |
| Quercetin | AR | −8.9 kcal/mol |
| Quercetin | EGFR | −8.2 kcal/mol |
| Quercetin | JUN | −9.2 kcal/mol |
| Quercetin | PIK3R1 | −9.7 kcal/mol |
| Quercetin | SRC | −8.7 kcal/mol |
| Quercetin | TP53 | −8.7 kcal/mol |
| Quercetin | VEGFA | −8.3 kcal/mol |
